# Management of chronic urticaria in children: a clinical guideline

**DOI:** 10.1186/s13052-019-0695-x

**Published:** 2019-08-15

**Authors:** Carlo Caffarelli, Francesco Paravati, Maya El Hachem, Marzia Duse, Marcello Bergamini, Giovanni Simeone, Massimo Barbagallo, Roberto Bernardini, Paolo Bottau, Filomena Bugliaro, Silvia Caimmi, Fernanda Chiera, Giuseppe Crisafulli, Cristiana De Ranieri, Dora Di Mauro, Andrea Diociaiuti, Fabrizio Franceschini, Massimo Gola, Amelia Licari, Lucia Liotti, Carla Mastrorilli, Domenico Minasi, Francesca Mori, Iria Neri, Aurelia Pantaleo, Francesca Saretta, Carlo Filippo Tesi, Giovanni Corsello, Gian Luigi Marseglia, Alberto Villani, Fabio Cardinale

**Affiliations:** 1 Clinica Pediatrica, Dipartimento Medicina e Chirurgia, Università di Parma, Parma, Italy; 2 Pediatric Unit, Maternal Infant Department, Azienda Sanitaria Provinciale Crotone, Crotone, Italy; 3 Dermatology Unit, Bambino Gesù Children’s Hospital, IRCCS, Rome, Italy; 4 Department of Pediatrics, Policlinico Umberto I, Sapienza University of Rome, Rome, Italy; 5 Generalist Pediatrician, Local Health Unit of Ferrara, Ferrara, Italy; 6 Primary care Pediatrician, Local Health Unit of Brindisi, Brindisi, Italy; 7 Pediatric Unit, Azienda di rilievo nazionale ARNAS “GARIBALDI”, Catania, Italy; 8 Paediatric Unit, “San Giuseppe” Hospital, Empoli, Italy; 9 Pediatric and Neonatology Unit, Imola Hospital, Imola, BO Italy; 10 FEDERASMA e Allergie Onlus – Federazione Italiana Pazienti, Prato, Italy; 11 Pediatric Clinic, Foundation IRCCS Policlinico San Matteo, University of Pavia, Pavia, Italy; 12 UO Allergologia, Dipartimento di Pediatria, Università di Messina, Messina, Italy; 13 Clinical Psychology Unit, Bambino Gesù Children’s Hospital, IRCCS, Rome, Italy; 14 Clinica Pediatrica, Department of Medicine and Surgery, University of Parma, Parma, Italy; 15 UOC Pediatria, Azienda Ospedaliero-Universitaria “Ospedali Riuniti”, Ancona, Italy; 16 Allergological and Pediatric Dermatology Unit, AUTC and University of Florence, Florence, Italy; 17 Department of Pediatrics, Senigallia Hospital, Senigallia, Italy; 18 Department of Pediatrics and Emergency, Pediatric Allergy and Pulmunology Unit, Azienda Ospedaliera-Universitaria “Consorziale-Policlinico”, Ospedale Pediatrico Giovanni XXIII, Bari, Italy; 19 UOC di Pediatria Azienda Ospedaliera “Bianchi-Melacrino-Morelli”, Reggio Calabria, Italy; 20 Allergy Unit, Department of Pediatric Medicine, Anna Meyer Children’s University Hospital, Florence, Italy; 21 Dermatology Unit, University of Bologna, Bologna, Italy; 22 Clinica Pediatrica, Azienda Ospedaliero-Universitaria di Parma, Parma, Italy; 23 Pediatric Department, AAS2 Bassa Friulana-Isontina, Palmanova-Latisana, Italy; 24 Pediatric Allergy Unit, Department of Medicine, Udine, Italy; 25 Clinica Pediatrica Università degli Studi di Palermo, Palermo, Italy; 26 UOC di Pediatria Generale e Malattie Infettive, Ospedale Pediatrico Bambino Gesù, Rome, Italy

**Keywords:** Allergy, Angioedema, Chronic urticaria, Chronic spontaneous urticaria, Children, Inducible uricaria, Management, Omalizumab, Pathogenesis, Pediatric, Therapy, Urticaria

## Abstract

The aim of this guidance is to provide recommendations to clinicians and other interested parties on chronic urticaria in children. The Italian Society for Pediatrics (SIP), the Italian Society for Allergy and Immunology (SIAIP), the Italian Society for Pediatric dermatology (SIDerP) convened a multidisciplinary panel that prepared clinical guidelines for diagnosis and management of chronic urticaria in childhood. Key questions on epidemiology, natural history, diagnosis, and management were developed. The literature was systematically searched and evaluated, recommendations were rated and algorithms for diagnosis and treatment were developed. The recommendations focus on identification of diseases and comorbidities, strategies to recognize triggering factors, improvement of treatment by individualized care.

## Introduction

Chronic urticaria (CU) is characterized by recurrent migrating skin lesions, called wheals or hives, angioedema (AE) or both lasting over 6 weeks. Wheals consist of a swelling area of different size and shape with a larger erythema, often pruritic. Lesions usually disappear in 24 h. In vasculitis and pressure urticaria lesions persist longer.

AE is a submucosal or subcutaneous swelling, and involves areas such as lips, eyelids, back of the hands and feet, scrotum. AE resolves in 1–3 days and causes pain, tingling, burning sensation or tension, but not itching. Prognosis *quoad vitam* and *quoad valetudinem* of CU is generally good. However, comorbidities can occur [1–4]**.** Overall, it is a condition of mostly unknown etiopathogenesis, and a frequent cause of specialist consultation and inappropriate diagnostic investigation, aimed at identifying a causal factor which is often not evident by history alone [4, 5].

Many guidelines on CU are available. None of them is dedicated to the paediatric population and some difference occurs among guidelines [6–11]. Many documents do not address specifically CU, whose etiopathogenesis is believed to be different from acute urticaria (AU), while many others discuss only chronic spontaneous urticaria (CSU) [10]. Therefore, the aim of the present guideline is to provide an evidence-based approach for the management of CU in children for use in clinical practice.

## Methods

In 2016, the Italian Society for Pediatrics (SIP), the Italian Society for Allergy and Immunology (SIAIP), the Italian Society for Pediatric dermatology (SIDerP) convened a multidisciplinary panel that included primary care paediatricians, general pediatricians, hospital paediatricians, allergist, immunologist, dermatologist, psychologist, methodologist skilled in systematic reviews and guideline. No conflict of interest was declared by panel members.

Team members defined the most relevant questions on CU in childhood, then they agreed on systematic literature research and literature evaluation strategy. Search strategy aimed at gathering studies, published from June 1, 2009 that is the last year included in the previous SIAIP/SIP/SIDerP guideline on CU [12], to January 1, 2018 concerning prevalence, incidence, aetiology, diagnosis, therapy, prognosis and psychological issues of CU in children.

The research was limited to studies published in English and Italian, with no preferential type of study.

In order to select the studies to be included in the final analysis, a hierarchic selection of literature sources was chosen, starting from secondary sources (evidence-based guidelines and systematic reviews) and proceeding with primary studies (RCTs and non-randomized trials).

Guideline documents were searched in the main general guideline websites and in the websites of the main scientific societies pertinent to CU. Systematic reviews were searched in Cochrane Database, DARE and Pubmed, using keywords as “urticaria” or “chronic urticaria” and “systematic review”. Evidence on primary studies was obtained by literature searches of PubMed/EMBASE. In PubMed, the following search strings and keywords were used - (“Epidemiology”[Mesh] OR “Causality”[Mesh]) OR “etiology”[Subheading]) OR “Prevalence”[Mesh]) OR “Cross-Sectional Studies”[Mesh]) OR “Incidence”[Mesh]) OR (“Diagnosis”[Mesh]) OR “diagnosis” [Subheading] OR (“Therapeutics”[Mesh]) OR “therapy” [Subheading] OR (“Prognosis”[Mesh] OR (“psychol*”[All Fields] OR “psychiatr*”[All Fields] OR “Depression”[Mesh] OR “Depressive Disorder”[Mesh] OR “anxiet*”[All Fields] OR “anx*”[All Fields] OR “Mood Disorders”[Mesh] OR “Affective Disorders, Psychotic”[Mesh] OR “Mental Disorders”[Mesh]) AND (“hives”[All Fields] OR “urticaria”[All Fields] OR “Urticaria”[Mesh] OR “Angioedema”[Mesh] OR “chronic urticaria”[All Fields] OR “chronic spontaneous urticaria”[All Fields] OR “chronic idiopathic urticaria”[All Fields] AND ((“2009/06/01”[PDAT]: “2017/12/31”[PDAT]) AND (“infant”[MeSH Terms] OR “child”[MeSH Terms] OR “adolescent”[MeSH Terms]) AND (English [lang] OR Italian [lang]).

Appropriate changes have been made to search in EMBASE: ‘chronic urticaria’/exp. AND ([english]/lim OR [italian]/lim) AND ([infant]/lim OR [child]/lim OR [preschool]/lim OR [school]/lim OR [adolescent]/lim) AND [humans]/lim AND ([embase]/lim OR.

Other studies, with no restriction of type were found through the electronic databases, references of the selected studies, hand searches or papers suggested by experts were also used.

Two authors have independently selected the studies relevant to each clinical question from the systematic research. They critically appraised each article, using the following validated tools when appropriate: SNLG criteria [13] and Grilli criteria [14] for Guidelines. AMSTAR tool [15] for Systematic reviews. AMSTAR-2 [16] was not used, as it has been recently published and its validity has not yet been verified. Assessment of Risk of Bias tool from Cochrane Collaboration [17] for randomized controlled trials (RCT). Newcastle-Ottawa scale for cohort studies, case-control studies and cross-sectional studies, when comparative [18] for observational studies. QUADAS-2 [19] for diagnostic studies. Users’ Guides to the Medical Literature [20] for prognostic studies. A complete list of appraisals of selected papers is available at http://www.siaip.it. Any disagreement in evaluation has been resolved via discussion. An evidence-based critical analysis was used to formulate conclusions and recommendations. Expert consensus was used when there was a lack of data. When it was possible, it was provided a recommendation based on grading of the quality of available evidence from the literature according to the Italian National Guideline (PNLG) method [13]. The criteria are as follows. Level of evidence. I. Evidence obtained from more than one properly designed randomized controlled trial and/or systematic revision of randomized study. II. Evidence obtained from one properly designed randomized controlled trial.III. Evidence obtained from non-randomized cohort studies with concurrent or historical controls, or their metanalysis. IV. Evidence obtained from retrospective case-control studies or their metanalysis. V. Evidence obtained from case-series with no control group. VI. Opinions of respected authorities, group of experts as shown in guidelines, consensus conferences or based on opinion of members of the panel of the current guideline. Strength of recommendation. A. Strong recommendation for performing diagnostic test or procedure, high quality evidence even not necessarily of level I or II. B. It is uncertain that diagnostic test or procedure should be recommended but the intervention should be carefully considered. C. Evidence not allowing recommendation for or against intervention. D. Performing diagnostic test or procedure is not recommended. E. Strong advisement against performing diagnostic test or procedure. Panel members reached agreement on levels of evidence and strength of recommendations. Before approval, the guideline was reviewed by nurses, parents, in order to consider the need for health and the expectations of affected children and their families, and experts who were identified by the panel. All comments were considered in the final document when appropriate. The recommendations in these guidelines will be disseminated through publishing articles and promoting courses. The impact of the current guideline on practice will be assessed by clinical studies. The guideline will be updated after 5 years to maintain validity.

*Question 1. What is the definition of CU, in the paediatric age?*

*Answer. CU in paediatric age is defined by the daily presence of wheals, that are not always associated with angioedema, for over 6 weeks or with brief periods of well-being due to therapy.*

Urticaria, AE or both are defined as chronic when they last for over 6 weeks. This definition allows to discriminate CU from AU, typically self-healing in a few days or weeks [7, 8], usually caused by viral infections or IgE mediated mechanisms. In a child with urticaria onset it is not possible to establish in which cases it will last over 6 weeks. In the paediatric population no markers have been identified [21]. Isolated AE is usually recurrent not persisting.

Thus far, there is no reason to believe that CU with AE is a different clinical entity from CU without AE, although some studies on adults seem to suggest that the presence of AE correlates with a higher chance of positive autologous serum skin test (ASST) [22, 23]. Conversely, isolated AE without urticaria often involves pathogenetic mechanisms and have clinical features that are different from AE associated with CU [24]. Therefore, it must be considered that isolated chronic AE without urticaria should be distinguished from CU, especially in the process of differential diagnosis.

*Question 2. What is the classification of CU?*

*Answer. CU in the child must be classified in “spontaneous” or “inducible”, in relation to the evidence of a triggering factor (Table 1).*

In CSU, there is no eliciting factor. In chronic inducible urticaria (CIU), one or more triggers, often physical agents, can be identified by history and/or laboratory tests [6, 8, 25]. Although “spontaneous” and “idiopathic” are often used as interchangeable terms, the definition of CSU is to be preferred as there is an anti-IgE autoantibody mediated form that should not be considered idiopathic. However, separating CU associated with anti-IgE autoantibodies from CSU [6] is not justified as many studies in adults have not found any histological differences between CSU and autoimmune CU [26]. Moreover, although some studies in adults found that autoimmune CU can have a more severe and prolonged course [27], there is no such evidence in children [4, 28]. Finally, in adults, data suggest that some forms of CIU may have an autoimmune mechanism [29, 30].

*Question 3. What is the prevalence and incidence of CU in the paediatric population?*

*Answer. Few data exist on epidemiology of urticaria in children, however it is reasonable to think that prevalence and incidence of CU in developmental age are both below 1% (Level of evidence IV).*

Few data are available on epidemiology of CU in children. Studies on mixed adult and children populations reported a lifetime prevalence of 0.8% [31]. A Korean survey on children aged 4-13 found a prevalence of 0,7%, with no difference among the two sexes [32]. Concerning incidence, an Italian study on children aged 0-14, where the diagnosis of CU was made by a paediatrician, showed an annual incidence of 0.6 to 2.1 /1000 children, and a prevalence fluctuating between 0,38% and 0,84% [33]. Overall, the prevalence of CU in children seems to be below 1%, and there is no significant difference among males and females [31–35].

*Question 4. What is the natural history of CU in paediatric age?*

*Answer. Remission at 3 years from onset of CSU in children happens in 30% to 50% of cases. Anaphylaxis is reported only in CIU (Level of evidence IV).*

Prospective as well as retrospective studies of good methodological quality on representative paediatric populations showed that chance of remission of CSU at a year from onset ranged from 10 to 32% [36–42]. At 3 years from onset, remission chance varied from 31 to 54% and at 5 years from 38 to 72%. The variability of the percentages was due to the different duration of the observation period, different criteria to define remission and sample size. A recent study reported a remission incidence of 10.3% per year. In the same study, a positive basophil activation test (BAT) or the lack of circulating basophils were associated to an almost double chance of remission after one year of follow-up [42].

The natural course of CU in children is therefore not different from adults [43–45]. However, some studies on adult and children samples have shown a higher probability of improvement of symptoms in subjects below 19 years old [46]. Female, age above 10 years and severe disease at onset have been related to a lesser chance of remission at 3–5 years of age [38–41].

The natural course of physical factors induced CU and cholinergic CU is not well known, but it is probably like CSU [47–51]. Some studies, mainly performed on adults, reported a more prolonged duration of disease in patients with cold urticaria [52, 53] and solar urticaria [54]. A longer persistence of CIU in atopic subjects compared to non-atopic ones has been found [47].

*Question 5. What is the etiopathogenesis of CU in children?*

*Answer. In most cases, CU in children is spontaneous and no external cause is found. However, in half of the cases of CSU, an autoimmune mechanism is possible. In a minority of patients, CU is associated with inducing factors, often physical (Level of evidence V).*

Pathogenesis of CU in children has been poorly investigated and studies have a low methodological quality. A systematic review [55] and subsequent studies [38, 41, 56] have showed that in most cases, an external cause of CU is not identified. In a recent survey, a potential cause has been found only in 8.8% of children with CU [41]. Most studies in children describe the frequency of factors associated with CU that were considered as causal agents, without being compared to a control population [36, 38, 41, 55–58]. Moreover, in most studies the association with a causal factor was established without evaluating the effectiveness of its removal (e.g. Infections, allergens). So, the prevalence of each potential causative factor varies among studies, even considering differences in setting and diagnostic criteria [55]. In CSU, an autoimmune pathogenesis has been reported in almost half of the cases [38, 41, 55, 56].

**•** *What is the role and the effect of inducing factors in children?*

*Answer. Inducing factors are the most frequent and often the only identifiable cause of CU in children (Level of evidence V).*

Inducing factors (Table 1) are usually the most frequent cause of CU in children [36, 42, 55, 56, 58, 59]. The evidence of the role of inducing factors in children’s CU has been confirmed by the reproduction of skin lesions when the relevant stimuli [25] are applied. In children, inducing factors triggered CU in 6.2 to 52.9% of cases [36, 55, 58–60]. In studies where inducing factors were investigated following international guidelines, the relative prevalence of CIU ranged from 22 to 40.1% of cases [42, 56]. The discrepancy of prevalence is due to heterogeneity of population samples and different diagnostic approaches. Dermographism, cholinergic urticaria and cold urticaria are the most common [42, 47, 55, 56, 61]. Different types of CIU can coexist in the same subject [25, 61, 62]. Moreover, in patients with CSU, hives may develop following exposure to physical stimuli, mainly dermographism and pressure [25, 61]. The pathogenesis of CIU is unclear. Serum from affected subjects (e.g. dermographism or cholinergic urticaria) injected into a monkey passively transfers the symptoms [63]. More recently, in patients with solar urticaria or cold urticaria, an IgE-mediated response against cutaneous, trigger-released auto-allergens seems to be implied [64–66]. Systemic symptoms such as bronchospasm, hypotension, loss of conscience, intestinal wall oedema, up to anaphylaxis and exitus may occur in solar urticaria, cold urticaria, pressure urticaria, cholinergic urticaria and aquagenic urticaria [29, 47–49, 52–54, 61, 64–69]. About 1/3 of patients with cold urticaria has had at least one episode of anaphylaxis, most commonly after bath in the sea or in the pool [52, 53, 65, 68]. In children, cold urticaria is rarely due to cryoglobulinemia or paraproteinemia [52].

**Table 1:** Classification of chronic urticaria

Clinical type	Subtype	Trigger/co-morbidities
Chronic Spontaneous Urticaria (CSU)	CSU with AE CSU without AE	• Autoimmunity • Infections • Others?
Chronic Inducible Urticaria (CIU)	CIU with AE CIU without AE	Physical • Aquagenic • Cold • Delayed pressure • Dermographism • Heat • Solar • Vibratory Non-physical • Cholinergic • Medications or allergens

**•** *What is the role of infections/infestations in children’s CU?*

Answer. The evidence on the role of viruses, bacteria or parasites in inducing CU is sparse and limited to single cases or case series. Few paediatric patients with CU and parasitical infestations that healed after the eradication of parasites have been described. This correlation has been occasionally reported in other infections (Level of evidence V).

Viral and bacterial infections have been reported to aggravate [6] or cause [4, 7, 8, 70] CU in children with a frequency ranging from 0 to 35% of patients with CU, while parasitical infestations from 0 to 37.8% [36–39, 41, 42, 55–57, 71]. The causal role of infections in patients with CU requires a high incidence of the infection in affected patients, and remission of symptoms after treatment [39, 57, 70, 71]. However, the prevalence of chronic infections in patients with CU is not different from the general population [8, 70]. Moreover, children with CU, affected by chronic infections or parasitic diseases, that are still symptomatic after eradication therapy have commonly been reported [42, 56]. These findings suggest that the association between infection and CU is mostly accidental [8] and that in many cases CU recovers because of the disease’s natural course rather than because of the treatment of the infection.

Bacteria are the most studied, particularly Helicobacter pylori. Protein components of *H. pylori* of molecular weight 21 and 35 Kd, can activate mast-cells in vitro, causing the release of histamine, TNF-alfa, IL-3, IFN-gamma and LTB4 [72]. Differences among studies in design and diagnostic methods make it challenging to interpret the association between *H. pylori* and CU. Furthermore, studies in children are few. A systematic review concluded that the chance of remission of CU in patients with *H. pylori* infection after eradicating therapy is significantly higher than in those who did not undergo eradication therapy or in those with CU without *H. pylori* infection [70]. A slightly more recent systematic review concluded that the evidence on benefits of eradicating therapy for *H. pylori* in CU was weak and conflicting [73]. Moreover, in a Turkish non-comparative study, performed on 222 children with CU, 32,8% of patients were tested positive to C13-UBT but only in one case a complete remission of cutaneous symptoms after eradicating therapy was observed [56]. Similar results have been reported in studies on smaller paediatric case series [28]. Even in adults, studies that show that eradicating *H. pylori* infection leads to the resolution or improvement of CU symptoms, are lacking [70, 74, 75].

Concerning other bacterial infections (e.g. Streptococcus, Staphylococcus, Chlamydia Pneumoniae), prevalence in CU does not differ from that in general population. Clinical trials either do not often clarify whether there was resolution of symptoms after the clearance of the infective agent [36, 58, 61, 63, 76] or they found that treatment did not resolve the disease [56]. For example, in a Turkish study on a large population of children, CSU resolved following antibiotic therapy in only one out of three patients with positive urine culture [41].

In children with CU and parasitic infection, anti-parasitic agents have been sporadically reported to improve cutaneous lesions in Western countries [77]. *Blastocystis hominis*, *Giardia lamblia, Dientomobea fragilis, Ascaris lumbricoides* and *Strongyloides stercoralis* have been frequently detected [41, 56, 57, 71, 78]*.* The incidence of parasitic infestations in children with CU varied from 0% to 37,8% [71]. In children, remission of CU after anti-parasitic treatment ranged from 0 to 100% of cases [39, 41, 56, 57, 71].

In the only study with a control group, the rate of resolution after anti-helminthic treatment was similar in children with and without parasitic infection [39]. Therefore, the relationship between CU and parasitic infestation in children remains unclear. A parasitic infestation may be considered a potential cause of urticaria in a few patients.

Non-controlled studies in adults have shown a high frequency of sensitization to Anisakis in patients with CU, with improvement of symptoms in a variable proportion of patients after a seafood-free diet [79, 80]. No data is available on the association between Anisakis infestation and CU in children.

Viral infections (Herpesviridae, HBV and HCV) have been identified as a cause of CU in anecdotal cases or non-controlled studies [58, 60, 81]. A possible role of latent infections by HHV-6 in adults has been suggested [82]. Up to date, however, there is no evidence of a role of viruses in CU in children.

**•** *What is the role of allergy in CU in children?*

Answer. There is no clear evidence that food allergens or medications provoke CU in children (Level of evidence V).

In children with CU, COX-1 inhibitors should not be used unless necessary because they could aggravate symptoms. (Level of evidence IV. Strength of recommendation D).

There is no evidence that IgE-mediated hypersensitivity reactions play a pathogenetic role in CU in children. Atopy is not predictive of severity or longer duration of CU in children [39, 41, 42, 83]. Nevertheless, a longer duration in atopic children with CIU has been reported [47].

### Contact allergy

Contrasting data have been reported on the role of contact hypersensitivity in CSU in adults [84–86]. Positive patch tests for common contact allergens have been shown in 42,9% of 543 patients, mostly adults (aged 5–85), with differences in sensitization due to age and occupation [86]. There is no evidence that avoidance of aptens can improve CU.

### Prevalence of atopic diseases

Adults with CU have a significantly higher prevalence of asthma, allergic rhinitis and atopic eczema compared to controls (10,8%, 9,8% and 19,9% vs 6,5%, 3,7% and 10,1% respectively) [83]. Similar trials are lacking in children. Prevalence of atopy, defined as positive skin test or personal history of allergic diseases, ranged from 13 to 35.9% in case series of children with CU [37–42, 60]. This confirms a similar frequency in a general paediatric population.

### Total IgE levels

In adults, levels of serum IgE are related to severity and duration of CU [87]. Higher levels of total IgE have been reported in children with CU than in those with AU, with no significant differences in inhaled or food allergens sensitisation and circulating eosinophils [88]. The meaning of this association is unclear.

### Food allergy

Despite parents’ views [4], food allergy is a rare cause of CU in childhood. In case series [28, 36–39, 41, 42, 56, 58, 59] of children with CU, the prevalence of food allergy varied from 0 to 8.6%. It must be pointed out that in many studies the oral food challenge (OFC) was not performed or, when performed, it was not double-blind controlled but open. So, reported rates are less reliable since CU is characterized by daily symptoms. Furthermore, in children with a positive OFC, the elimination diet did not always cure urticaria [28, 37, 39, 41, 57, 89]. In a mixed adult and children population, the rate of IgE mediated food allergy ascertained by open OFC was 2,8% [90]. Studies in adults but not in children show a possible correlation between IgE to lipid transfer protein and CU [91, 92].

### Aeroallergens

Although, it has been shown that aeroallergens may trigger CU [59], there is no evidence of an association between IgE mediated sensitization to inhaled allergens and CU in children.

### Medications

Medications causing CSU in children have been sparsely reported [38, 59]. In a large population of children with CSU, no suspected drug allergy was confirmed [41].

Regarding NSAIDs, COX-1 inhibitors, even the ones that are not related to one-another, can exacerbate CU through non-immune-mediated mechanisms [8, 92] independently from a causing role [93, 94]. In children with CSU, single blind oral challenge with ASA was positive in 24% of cases, and lip angioedema was the more common manifestation [93]. CU appears also to be the main risk factor for NSAIDs hypersensitivity in childhood [94]. Therefore, it is advisable not to give NSAIDs to children with CU unless necessary.

**•** *What is the role of pseudo allergens and vasoactive amine rich foods in children’s CU?*

Answer. There is insufficient evidence that pseudo allergens and vasoactive amine rich foods can modify CU’s course (Level of evidence V)

Intolerance to additives has been associated to CU in 2,6 to 21% of children in low quality studies that did not report whether a low additive diet improved symptom [55]. In 81% (13/16) of children with idiopathic CU, symptoms recovered after a 3-week low pseudo allergen diet; only 6/13 patients underwent a double-blind OFC with suspected additives, which was positive in 5/6 cases [95]. In 100 patients with CU aged between 14 and 67 years old, two adults did not pass single blind OFCs to 11 additives including food colourings and preservatives [96]. These two patients passed a double-blind controlled OFC to the same additives. In an open study in adults, a low pseudo allergen diet improved CSU in around a third of the patients [97]. Limitations of the study included lack of a control group and missing evaluation of reintroduction of excluded foods. The same methodological bias had an open study in adults that showed the effectiveness of a 3–4-week low vasoactive amine diet in 75% of patients [98]. In conclusion, existing data do not support the causative role of pseudo allergens in CU.

**•** *What is the role of autoimmunity in children’s CU?*

Answer. *In 30-50% of children with CSU, autoimmune mechanisms are probably implied (Level of evidence IV). We can hypothesize the role of auto-allergens in some forms of inducible urticaria (Level of evidence V). In contrast to adults, the paucity of studies on autoimmune diseases allowed to associate children’s CU only with anti-thyroid antibodies, autoimmune thyroiditis and coeliac disease (Level of evidence V).*

CSU is often associated with autoimmune thyroiditis and coeliac disease in children [1, 2]. Large longitudinal studies in adults show that co-morbidity of CU and autoimmune diseases is common [99]*.* There is accumulative evidence that a causal relationship between type I autoimmunity and CU is “suggestive” and between type II autoimmunity and CU is “probable” [100].

### Serum autoantibodies activating mast cells and basophils

The presence of circulating IgG autoantibodies against high affinity receptor for IgE (FcεR1α) or anti-IgE antibodies (type II autoimmunity) that can release mediators from mast-cells and basophils is well established in many patients with CSU [100, 101]. Functional tests used to detect these autoantibodies include in vitro tests, such as basophil histamine release assay (BHRA) and basophil activation test (BAT)*,* as well as in vivo tests, notably autologous serum skin test (ASST) and autologous plasma skin test (APST). In vitro and in vivo tests are not interchangeable and study different pathogenetic mechanisms of the disease [102, 103]. A positive ASST has been reported in 22 to 53.5% of children with CU [28, 37–39, 41, 58]. ASST can be positive even in healthy subjects or in patients affected by different diseases. Only a subgroup of patients with positive ASST has a positive in vitro histamine releasing test [101–103]. It has also been noted that ASST uses non-purified IgG. A positive ASST result persists after removal of complement protein and IgG adsorption [104]. Therefore, ASST indicates a mast cell activation induced not only by autoantibodies but also by other serum factors that can promote histamine release [105]. There is no difference in the frequency of positive ASST between children with CU affected by parasitic infestation and those without infestation [57]. In adults with CU, APST is more frequently positive than ASST [102]. There is no experience in children about the use of APST.

Regarding in vitro tests, histamine releasing IgG anti-FcεR1α functional antibodies have been documented in 47% of children with CU compared to 0% of controls with atopic eczema [37]. Other studies reported significantly higher levels of BAT in children with CSU compared to healthy controls [106]. In these studies, there was an overlapping of values between the two populations, so that it was not possible to identify a cut-off value to separate them [42].

Autoantibodies have been detected by Western blot method or ELISA immunoenzymatic method in adults with CU [100], but not in children. Regarding Witebsky’s criteria, that are necessary to define CU as a type II autoimmune disease, direct evidence and circumstantial evidence, drawn from clinical practice, are not complete and an animal model is lacking [105]. Some forms of CIU (solar, cholinergic, cold) may involve the production of IgE against auto-allergens expressed in the skin as an effect of thermic stress or other physical factors, as shown by positive passive transportation test [29, 64–66].

### Thyroid autoimmune disease

Patients with CU are at risk of thyroid autoimmune disease (particularly Hashimoto thyroiditis). Case-control studies show that in children with CSU the prevalence of autoimmune thyroiditis is 10 to 30 times higher than in a general population [1]. Levels of IgG anti-thyroid antibodies are significantly increased in patients with CU compared to controls; these levels are also higher in adults than in children [107]. It is unclear whether anti-thyroid antibodies play a pathogenic role. Higher anti-thyroperoxidase IgE levels were found in adults with CSU, making it possible to hypothesize auto-allergy mechanisms [108]. The presence or absence of antibodies do not confirm nor exclude the diagnosis of thyroiditis [109] and up to date a causal role of thyroid disease on CU onset has not been proven unequivocally [110]. In adults, thyroid diseases are often associated with CU, while in children the prevalence of hypothyroidism, often due to Hashimoto thyroiditis more than Graves’ disease, is below 1% and hyperthyroidism has not been reported [55, 107]. There is no clear evidence that in patients with thyroid autoimmunity, CU has a different course or that treatment with thyroid supplementation therapy improves urticaria.

### Coeliac disease

Case reports and case control studies have shown the association between CU and coeliac disease in children as well as adults [2, 99]. The prevalence of coeliac disease in patients with CSU varies among studies, and it is increased by 8–10 times when compared to a general population [2]. A remission of cutaneous symptoms after a gluten free diet has been also reported [2]. Conversely, studies on large populations highlighted a slightly higher prevalence of CU as well as AU in patients with coeliac disease in comparison to healthy controls [99, 111].

### Other autoimmune diseases

Adults with CU seems to have an increased risk of developing other autoimmune diseases compared to children, possibly because incidence of autoimmune diseases rises with age. In children with systemic lupus erythematosus, CU is rare (0–1% of cases) when compared to adults [99, 112]. Few cases of systemic lupus erythematosus in children with CU have been described [112]. The presence of anti-nucleus and anti-DNA antibodies without connective tissue disorders has been rarely observed in children with CU [28, 55, 56]. The prevalence of rheumatoid arthritis, Sjogren syndrome, type 1 diabetes is increased in adults with CU. In childhood data are lacking [56, 99, 113]. Vitiligo, pernicious anaemia and Raynaud’s phenomenon with anti-centromere antibodies have been sparsely reported in children as well as in adults [56, 99, 113].

What is the role of the activation of coagulation and fibrinolysis in CU in children?

Answer. Insufficient data have been reported on the role of coagulation and fibrinolysis processes in the pathogenesis of CU in children (Level of evidence V).

Studies on adults have shown that the coagulation 7cascade can have a role in the pathogenesis of CU. The cascade seems to be initiated by expression of tissue factor by activated eosinophils and the release of thrombin. In animal models, thrombin increased vascular permeability with a direct action on endothelial cells as well as an indirect one on histamine and other mediators released by mast-cells [103]. During urticaria exacerbations, adults with CU showed increased prothrombin fragments serum levels [1, 2, 102]. In adults with CU, there was fibrinolysis [114, 115]. Serum levels of D-dimer and fibrin degradation product increased during exacerbations of CU in adults and they have been proposed as markers of severity and response to antihistamines [115, 116]. The activation of the processes of coagulation and fibrinolysis in CU in paediatric age is supported by few studies on mixed adult and paediatric populations [115] and by a Japanese study that showed the increase of serum levels of prothrombin fragments 1 and 2 in the small group of children with CU [117].

*Question 6. Is CU in children associated to other organ diseases or systemic diseases more frequently than in non-selected population?*

*Answer. There is no evidence of an association of CU in children and other organ or systemic diseases (Level of evidence V)*

Although in adults it has been reported that CU is associated with rheumatic, inflammatory and psychiatric diseases [118], irritable bowel disease [119], cancer [120] and metabolic syndrome [121], the evidence is insufficient. There are no similar studies on children. Constipation and irritable bowel are not more frequent in children with CU [122].

*Question 7. Can psychological factors determine CU or exacerbate it?*

*Answer. Studies performed on adults might suggest a role of psychological factors in the development or exacerbation of CU. In small populations of children, weak data seem to support this hypothesis (Level of evidence IV).*

Many studies suggested that psychological factors might contribute to the development or the exacerbation of CU, supposing that they could play a role in its pathogenesis. Some authors suggested an interaction between nervous and immune systems [123]. Animal models have pointed out that acute stress caused the activation of skin mast-cells and the expression of corticotrophin releasing hormone receptors [124]. Adults with CU had significantly higher scores in tests to diagnose obsessive-compulsive disorders, depression, anxiety, insomnia, stressing events than controls [123]. In adults, numerous studies have been performed [6, 123, 125], but in children data are still few. In 27 children with CU, there was a higher prevalence of psychiatric disorders (70% vs 30%), mainly anxiety and depression, but also separation anxiety, specific phobias, psychosomatic disorders than in controls [126]. No correlation has been found with severity or duration of disease. About 2/3 of children underwent a stressing event in the 6 months before the onset of CU. More trials are needed to clarify the role of psychological factors in causing or aggravating CU, and the efficacy of a multidisciplinary approach with appropriate psychological and pharmacological support.

*Question 8: Can clothes or temperature changes worsen CU?*

*Answer. There are no studies that document the role of clothes and temperature on the course of CU in children, excluding subjects with cold-urticaria, heat-urticaria, cholinergic urticaria (Level of evidence VI).*

### Diagnostic work-up

The aim of the diagnostic work-up is to establish criteria to recognize patients with urticaria, make a differential diagnosis, identify triggering factors, assess disease activity and its control.

*Question 9. Which are the criteria that allow to diagnose CU in children?*

*Recommendation. The diagnosis of childhood CU is based on appearance of itchy wheals, not always associated with AE, persisting daily or on most days for at least 6 weeks. No laboratory test is needed to diagnose CU (Level of evidence VI. Strength of the recommendation A)*

The diagnosis of CU is based on history and occurrence and duration of wheals, typically itchy, migrating, fading with finger pressure. The duration of a single lesion is usually less than 24 h with episodes lasting over 6 weeks. AE is characterized by non-erythematous oedema, associated to a burning or pain sensation lasting up to 72 h, often located in the face, genitalia and extremities. There is no instrumental or laboratory test to diagnose CU.

*Question 10. Which conditions should be considered in the differential diagnosis of CU and what are the clinical or laboratory criteria that help in the differential diagnosis? Are vasculitic urticaria, monogenic syndrome associated urticaria and bradykinin mediated AE different clinical entities from common CU?*

*Recommendation. Differential diagnosis is necessary in any case of CU as wheals can be found in many acquired or hereditary conditions, with different pathogenic mechanisms, such as papular urticaria, mastocytosis, some vasculitis and genetic syndromes. Wheals must also be differentiated from other elementary lesions, as papulae. Recurrent isolated AE should be distinguished from bradykinin mediated angioedema, hypoproteinemic oedema and some cancers. An evaluation of morphology of lesions, duration and associated signs and symptoms leads toward a diagnostic hypothesis that must be confirmed or not by diagnostic tests listed in Table 2 (Level of evidence VI. Strength of recommendation A).*

CU should be distinguished from many genetic or acquired diseases, based on various clinical characteristics and the result of diagnostic tests [127–135] (Table 2). Vasculitic urticaria and monogenic syndrome associated urticaria can be differentiated from common CU because of their different macroscopic appearance, histology, clinical evolution of lesions and response to therapy.

**Table 2:** Differential diagnosis of chronic urticaria

Disease	Clinical criteria	Laboratory/instrumental data	Diagnostic criteria
Mastocytosis [127–129]	-Maculae, round or oval papulae, brownish, from few mm to 2 cm diameter. Lesions become erythematous and swollen after mechanical stimulation (Darier’s sign). -Asymptomatic, rarely mild itch. -Systemic forms are associated with flushing, wheezing, abdominal pain, diarrhoea, syncope.	-Possible elevated serum tryptase in intercritical phase (systemic mastocytosis). -Possible eosinophilia (cutaneous and systemic mastocytosis). -Possible cytopenia (systemic mastocytosis).	-History. -Clinical features; Darier’s sign. -In rare cases, skin biopsy.
Papulous urticaria (strophulus; children papulous dermatitis) [130, 131]	-Erythematous papulae of few mm diameter on exposed areas (face, limbs), isolated or confluent, sometimes with vesicles on top, rarely itchy. Long persistence. Spontaneous resolution. [128]	None.	-History, contact with agents in gardens, fields, animals etc. -Clinical features.
Vasculitic urticaria [132, 133] Normocomplementemic	-Papulous erythemato-purpuric lesions that do not fade with finger pressure, lasting over 24 h. Pain and/or burning feeling, sometimes itch. It can be associated with fever, arthralgia, petechiae. Lesions resolve with secondary hyperpigmentation.	-Blood cell count. -Increase of inflammatory markers, ANA, anti-DNAds antibody, rheumatoid factor positivity. -Mutation of IL3 DNASE.	-History. -Skin biopsy (leukocytoclastic vasculitis).
Vasculitic urticaria [132, 133] Hypocomplementemic	-Fever, arthralgia, petechiae. -Association with systemic lupus erythematosus.	- Hypocomplementemia (C1q, C3, C4). -Elevated ESR. -Positive ANA, anti-DNAds antibody, rheumatoid factor.	History. Urticaria for over 6 months. -Systemic symptoms. -Skin biopsy (leukocytoclastic vasculitis).
Mc Duffie syndrome with anti-C1q antibodies	-Urticaria for over 6 months associated with arthritis, arthralgia, lung pathology, uveitis, episcleritis, glomerulonephritis.	-C1q, C3, C4; anti-C1q autoantibodies.	-History. -Urticaria for over 6 months. -Skin biopsy (leukocytoclastic vasculitis). -Anti-C1q autoantibodies.
Criopirinopathy (CAPS) [134]: 3 different phenotypes -Familial cold autoinflammatory syndrome (FCAS) -Muckle-Wells syndrome -Neonatal onset multisystemic inflammatory disorder (NOMID) or chronic infantile neuro-cutaneous articular syndrome (CINCA)	-Autosomic dominant, de novo mutations described. -Early onset in the first months of life. -Plaques, erythema or papulae that disappear in 24 h; no itch. -Exanthema, fever, arthralgia and conjunctivitis after 1–2 h of exposure to cold, lasting < 24 h. -Arthralgia or periodic arthritis, conjunctivitis, secondary generalized amyloidosis, neurosensory deafness. -Maculo-papular wheal-like eruption, non-constant fever, failure to thrive, neurosensory progressive hypoacusis, uveitis, optic neuritis to blindness, variable articular symptoms, non-foreseeable defects of long bone growth, chronic meningitis, chronic headache, intellectual disability.	-Increased ESR, CRP, anaemia, neutrophilic leucocytosis, absence of autoantibodies. -Negative response to cold challenge.	-More than 2 markers among: urticarial rash; exacerbations with cold/stress; neurosensory hypoacusis; arthralgia/arthritis, myalgia; chronic aseptic meningitis; skeletal abnormalities (overgrowth of epiphysis and frontal bone) WITH: increase of inflammation markers; serum A amyloid. -Molecular diagnosis (NLRP3, NLRP12);
Tumor necrosis factor (TNF) receptor 1 associated periodic syndrome (TRAPS)		-Neutrophilic leucocytosis, increased ESR and CRP, A amyloid serum; soluble TNF receptor.	-History. Mutation of the TNFRSF1A gene.
Bradykinin mediated angioedema [135]	-Dominant autosomic or de novo mutation. -Isolated angioedema lasting more than 24 h; no itch; sometimes gastrointestinal or respiratory involvement. -No response to anti-histamines. -Two different types, a most frequent one caused by C1-INH quantitative defect, the other by functional impairment.	-Decrease of C4 levels. -Reduced C1-INH in the first type. -C1-INH and C4 levels reach adult levels at 2–3 years old.	-History. Intake of ACE inhibitors. -C4 determination. -Quantitative and functional determination of C1 INH. -Mutations of SERPING 1 gene.
Hypoproteinemic oedema	-Peripheral swelling, serous cavities effusion. Diarrhoea, poor growth. Nutritional deficit.	Serum protein electrophoresis.	-History. -Systemic symptoms. -Hypoalbuminemia.
Head and neck tumors, lymphoma	Local swelling.	-Imaging (RX, CT, MRI). -ESR, LDH, Serum protein electrophoresis.	-History. -Biopsy.

*Question 11. What is the role of history and physical examination in identifying the aetiology of CU in children?*

*Recommendation. History and physical examination are the guide to identify a possible underlying cause of CU and decide whether other diagnostic tests are needed (Level of evidence V. Strength of recommendation A).*

History is the first step in the diagnostic process of CU [6, 8, 25, 55]. Clinical history is helpful to differentiate CSU from CIU and to identify a specific cause [25, 56]. Clinicians should investigate:

- Frequency and duration of skin lesions. Wheals lasting over 24 h lead to delayed pressure related CU or vasculitic urticaria. On the contrary, wheals lasting less than an hour are common in physical urticaria (except for pressure induced urticaria).
- Shape, dimension, distribution of wheals.
- Presence of isolated or associated angioedema.
- Family history of atopy, urticaria, systemic disorders.
- Age at onset of symptoms.
- Triggering and aggravating factors, particularly food habits, medications, physical exercise or physical factors, supposed interval between exposure a wheal appearance.
- Circumstances and places when symptoms occur (night/day, inside/outside, free time...).
- Systemic signs and symptoms that suggest organ or systemic diseases, such as coeliac disease, vasculitic urticaria or auto-inflammatory conditions such as periodic cryopyrin-associated syndromes [134, 136, 137].
- Subjective symptoms, as pain, burn, itch.
- Quality of life.
- Former tests executed.
- Effectiveness of present or past treatment.

Any laboratory test should be performed when history and clinical data suggest an eliciting factor or a systemic disease to confirm its role in the pathogenesis [138, 139].

*Question 12. In case of suspected CIU, is it necessary to perform diagnostic tests for inducible urticaria?*

*Recommendation. Specific tests should be used to confirm the suspect of inducible CU (Level of evidence V. Strength of recommendation B).*

Specific tests (Table 3) should be performed to confirm the suspicion of inducible urticaria and when possible, to determine minimal stimulation cut-off, useful to define the activity of disease and response to therapy [7, 25, 140, 141]. It must be underlined, however, that in 1/3 of cases tests result negative. Different types of inducible urticaria can co-exist in the same subject; in this case the various triggers should be tested in sequence [142, 143]. To make the tests more reliable, anti-histamines and corticosteroids should be interrupted 3 and 7 days before the test respectively. Stimuli should be applied to parts of the body that were not involved by urticaria in the last 24 h, to avoid a reduced response due to temporary local refractoriness.

**Table 3:** Diagnostic tests for CIU [7, 25, 140, 141]

Type of urticaria	Site	Test	Time to read results
Dermographism	Volar surface of the forearm or superior surface of the back	Rub with a blunt smooth object (pen, dermatographometer, 36 g/mm^2^)	10 min
Cold urticaria	Volar surface of the forearm	Ice cube in a plastic or a thin film for 5 min; TempTest®	10 min
Heat urticaria	Volar surface of the forearm	Heat source at 45 °C (es: Temp*Test*®, tube with hot water) for 5 min	10 min
Delayed pressure urticaria	Back or thigh or volar surface of the forearm	Weight over arm or shoulder (7 kg backpack) for 15 min. Dermographometer 100 g/mm^2^ for 70 s in research	6 h (0.5–12 h)
Solar urticaria	Covered skin: glutes	UVA (6 J/cm^2^), UVB rays (60 mJ/cm^2^), direct light	10 min
Vibratory urticaria/angioedema	Volar surface of the forearm	Vortex for 5 min at 1000 rpm	10 min
Cholinergic urticaria		1. Exercise test (free run or tapis roulant or cyclette) for 15–30 min. 2. Immersion of body or arm in water at 42 °C for ≥15 min after increasing body temperature ≥ 1 °C higher than basal temperature	During the tests and 10 min after the end
Aquagenic urticaria	Trunk	Compress with 35–37 °C water for 20–30 min	At the end of the test
Contact urticaria	Back or forearm	Skin prick test Patch test	15 min 15 min-48-72 h

*Question 13. When clinical history does not indicate an underlying cause, is it recommended to perform laboratory tests to identify allergic or infective triggers, in a child?*

*Recommendation. When clinical history does not suggest a temporal relationship between exposure to an allergen and onset of symptoms, it is not recommended to perform allergy tests to foods, additives, inhaled particles or medications (Level of evidence VI. Strength of recommendation D).*

*When there is a history of cause-effect relationship between exposure to an allergen and occurrence of urticaria and IgE tests are positive to the relevant allergen, diagnosis can be ascertained by effectiveness of allergen avoidance and positive provocation test to the same allergen (Level of evidence V. Strength of recommendation A).*

*Diagnostic tests for infectious disease should be performed only when there is a suspicion based on clinical history or laboratory tests (Level of evidence V. Strength of recommendation B).*

An IgE mediated reaction to foods or medications can be considered a potential cause of CU when the reaction develops within one or two hours following allergen exposure and it vanishes in a few hours. Regarding allergic reaction to NSAIDs, they can occur within 24 h. If the interval time between allergen exposure and urticaria occurrence is different, IgE mediated reactions are ruled out and allergy tests (skin prick test [144], serum specific IgE, challenge) to foods [145] and medications [146] should not be performed. Patch tests to foods are not recommended [147].

Additives, preservatives and colouring free diet in foods and medications should be advised only in the rare cases in which we can suspect a relationship between their intake and the onset of symptoms. If the diet is effective, a double-blind placebo-controlled provocation test is needed to firmly establish the diagnosis.

The rate of resolution of CU after the eradication of infectious agent is low [4, 8, 25, 41, 55, 56, 71, 78, 96]. Therefore, viral, bacterial and parasitic infections must be investigated only in patients with suggestive history or laboratory tests. There is weak evidence that laboratory testing for parasites should be performed in patients with history of abdominal pain [56, 78], earlier infestations, staying in regions at risk, unexplained eosinophilia [71]. In patients with CU a parasitic infestation is not associated with AE, total IgE levels, high CRP, positive prick test, positive ASST [56].

Question 14. Is it useful to perform Autologous Serum Skin Test (ASST) in the diagnostic workup of CU?

Recommendation. ASST should be considered a screening test for autoantibodies (Level of evidence IV. Strength of recommendation B). ASST should not be routinely performed in children with CU (Level of evidence IV. Strength of recommendation D)

Functioning circulating IgG antibodies against high affinity receptor of IgE (Fc (epsilon) RI (alfa) receptor), and against IgE themselves, can be measured in vitro by BHRA or by BAT, which are both poorly standardized methods. It is also possible to use Western Blot or ELISA, non-commercialized immunoassay, which are expensive, have little specificity and sensitivity and do not differentiate between functional and non-functional autoantibodies [148, 149]. In vivo, ASST showed lower diagnostic accuracy when compared to BHRA [37] since it contains both IgG and serum factors that can promote histamine release. Therefore, the test must be considered an expression of auto-reactivity, and not of the presence of functional autoantibodies.

Negative ASST excludes an autoimmune pathogenesis even in patients with positive BHRA or BAT. In children, there is a concordance of 83% between ASST and BHRA [37, 58]. None of the proposed tests allows to formulate a certain diagnosis of autoimmune CU and it has been proposed as diagnostic gold standard the presence of a positive biological test (BHRA, BAT with CD63 expression), of ASST and of an enzymatic immunoassay [105]. From a clinical point of view, in children with CU, there are contrasting data on the association of positive ASST and CU severity [150, 151], time course [39] or response to treatment. In adults, it is unclear whether ASST negativization occurs when CU resolves [152–155]. Therefore, ASST should not be routinely performed. In BAT, high levels of expression of CD63 are associated with a higher urticaria activity score 7 (UAS7), although with low sensitivity and specificity [106].

Question 15. Is it useful to perform tests to rule out coeliac disease, thyroiditis, other autoimmune or neoplastic disorders in children with negative history and physical examination?

*Recommendation. Children with CU should be screened for coeliac disease and thyroid diseases (Level of evidence V. Strength of recommendation B) but not for other autoimmune diseases or malignancies (Level of evidence V. Strength of recommendation D).*

CU in children is rarely associated with hypothyroidism [55, 107, 109, 110], anti-thyroid antibodies [1, 112], or coeliac disease [111]. Coeliac disease can cause CU2. Laboratory tests to identify these conditions should be obtained in all patients, even when specific symptoms are lacking [99]. It has also been advised to monitor patients with CU because they can develop hypothyroidism or anti-thyroid antibodies [8] over time.

In childhood, it is not adviseable to investigate autoimmune diseases or cancers, since case reports have been hardly reported [28, 55, 56, 112, 113].

Question 16. Which diagnostic workup is appropriate for children with CSU?

Recommendation. In children with CSU with negative history and physical examination, it could be considered to perform blood tests for inflammatory diseases (blood cell count, CRP, ESR (Level of evidence V. Strength of recommendation B), and to test for autoimmune diseases (coeliac disease, thyroiditis) (Level of evidence V. Strength of recommendation B.)

In the diagnostic workup of CSU (with or without AE), history and physical examination are the basis to establish the need to perform laboratory tests and to choose their sequence. If history and physical examination are negative, laboratory tests are rarely useful [138, 139, 156]. Diagnostic testing for autoimmune diseases, associated to CU, can be performed.

**Fig. 1:**
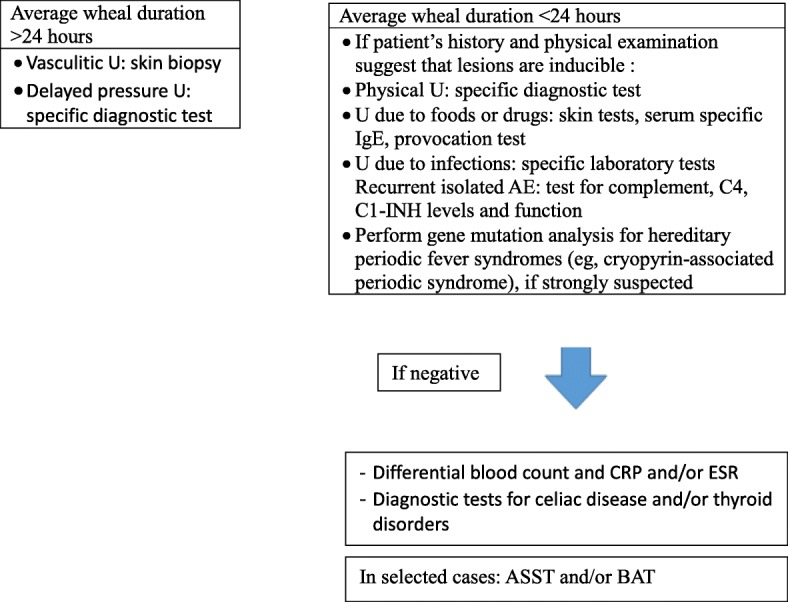
Algorithm for diagnosis of subtypes of CU

This task force proposes, therefore, a simple diagnostic workup (Fig. 1).

1. If a single wheal lasts over 24 h, and delayed pressure urticaria is ruled out, a skin biopsy can be necessary to confirm a diagnosis of vasculitic urticaria.

2. If a single wheal lasts less than 24 h, different possibilities must be considered.

a) If history or clinical features are suggestive for underlying causes (physical factors, medications, foods, additives, infections, autoimmune diseases) specific diagnostic tests should be performed. Dermographism, however, should be searched in all children with CU.

b) In case of recurrent, isolate AE, without any clinical feature or history of associated diseases, hereditary AE should be ruled out [157].

c) When there is a suspicion of a genetic disorder, cryopyrin gene should be analysed.

d) In the remaining cases, children might undergo additional tests including blood cell count, ESR, CRP to reassure parents on the benignity of the clinical condition; FT4, TSH, anti-microsome antibodies, anti-thyroglobulin and anti-thyroperoxidase antibodies, DGP-AGA (under 2 years of age), anti-TTG, IgA to identify the association with autoimmune diseases.

e) ASST and BAT should not routinely be performed to better understand the pathogenesis or for research purposes.

*Question 17. Is it advisable to use severity scores in children with CSU?*

*Recommendation. Currently there are no validated severity scores for CSU in paediatric age. However, in clinical practice it is possible to use adult scores (*Urticaria Activity Score 7-*UAS 7) to rate the severity of disease and to assess the response to treatment (Level of evidence V. Strength of recommendation B).*

The severity of CU should be evaluated in daily practice as well as in clinical trials. Currently there is no severity score that is validated for CU in children. Urticaria Activity Score (UAS7) [7] is the most used score to determine disease activity, its impact on quality of life and response to therapy. Some authors proposed its use in the child [42], even adapting it to body surface [158]. UAS7 is the sum of daily symptoms’ scores during a period of 7 consecutive days. It is requested to the patient to fill a sheet where he daily records the severity of itch and the number of wheals for 7 days [7]. UAS7 allows then to categorise the severity of CSU in severe (28–42), moderate (16–27), mild (7–15), well controlled (1–6), absent (0), and to define response to treatment (Table 4). UAS7 should be checked at follow-up visits.

**Table 4:** Weekly Urticaria Activity Score (UAS 7) [7, 158]

DAY	SCORE WHEALS/24H				SCORE ITCH/24H				SUM
	None	< 20	20–50	> 50	Absent	Mild	Moderate	Intense	
1	0	1	2	3	0	1	2	3	… ..
2	0	1	2	3	0	1	2	3	… ..
3	0	1	2	3	0	1	2	3	… ..
4	0	1	2	3	0	1	2	3	… ..
5	0	1	2	3	0	1	2	3	… ..
6	0	1	2	3	0	1	2	3	… ..
7	0	1	2	3	0	1	2	3	… ..

UAS7 has some weaknesses. It is based on self-evaluation only; being a prospective score, it cannot be used during the first evaluation of the patient; its evaluation is difficult if the patient forgets to mark the score on some of the days. Other scores have been validated in adults: Angioedema Activity Score to evaluate AE [159], Urticarial Control Test to evaluate the control of the disease [160, 161].

### Treatment

The first goal of treatment of urticaria is to control symptoms by avoidance of the triggering factor. When this is not possible, the approach to treatment of CU requires a symptomatic medication.

*Question 18. Can treatment of autoimmune thyroiditis or coeliac disease cure CU?*

*Recommendation. There is no clear evidence that treatment of autoimmune thyroid disease or coeliac disease associated with CU can have an impact on natural course of CU. However, in clinical practice the treatment is advisable (Level of evidence V. Strength of recommendation B).*

*(Level of evidence V).*

The hormone replacement therapy, used in patients with hypothyroidism, can positively affect CU [107]. In case of euthyroidism, even in presence of anti-thyroid antibodies, the treatment with L-Tiroxine is not advised [6], and thyroid monitoring should be continued. The resolution of CU during a gluten free diet has been sporadically observed in patients with coeliac disease [2].

*Question 19. Is it advisable to start an additive and/or pseudo allergen free diet in the child with CU?*

*Recommendation. When history is negative, children should not go on an additive and/or pseudo allergen free diet (Level of evidence V. Strength of recommendation E).*

Studies [55] on the efficacy of a pseudo allergen free diet, including additives and preservatives, in CU are few and performed on mixed case series of adults and children. These studies did not provide evidence that these interventions are effective when history is negative.

*Question 20. What is the drug of choice for CU?*

*Recommendation. Second-generation H1-antihistamines are the first-choice treatment for CU (Level of evidence I. Strength of recommendation B).*

Second (new)-generation H1-antihistamine are the first option in the treatment of CSU. A recent review of 73 studies with 9759 participants, including adolescents over 12 years of age, although none of them included specific paediatric data, concluded that anti-H1 anti-histamine drugs are beneficial in less than 50% of cases [162]. More recently, a blind randomized controlled trial, performed on mixed adult and adolescent populations, confirmed the efficacy of cetirizine (10 mg), fexofenadine (180 mg), bilastine (20 mg), desloratadine (5 mg), ebastine (20 mg) [163]. In a non-controlled prospective study in subjects with AU or CU aged 11 to 92 years, levocetirizine 5 mg daily for 2–6 weeks greatly improved or resolved symptoms in 60–80% of patients. Overall, 50–74% of patients perceived improvements in quality of sleep/daily activities and 50–65% of patients rated the onset of action for levocetirizine as very rapid or rapid [164]. In a comparative double-blind placebo-controlled trial [158], in subjects aged 2–11 years old, no significant difference has been found between desloratadine and rupatadine in wheal reduction. However, rupatadine, but not desloratadine, reduced the itch significantly compared to placebo. Quality of life was significantly better in patients treated both with rupatadine and with desloratadine. No difference was found in incidence of adverse effects between active groups and placebo group.

Second generation H1 antagonists are generally well tolerated [158, 162, 163, 165–168], except for astemizole and terfenadine whose metabolism by P450 liver cytochrome can be blocked by the concomitant administration of ketoconazole or erythromycin, causing cardiotoxic effects.

H1-antihistamines should be given for 1–2 weeks and, if effective, the need to continue should be re-evaluated every 3–6 months. Second generation H1 antagonists, approved for paediatric use, are listed in Table 5. Levocetirizine, active enantiomer of cetirizine, has been approved by FDA to treat non-complicated CSU in children from 6 months of age [166]. Earlier long-term studies have shown a good safety and tolerability profile of cetirizine and levocetirizine administered at a double dose in children aged between 12 and 24 months old, suffering from atopic dermatitis [169, 170]. Bilastine has a good tolerability and safety profile in children aged 2 to 12 years old suffering from CU [167]. It is desirable to have more clinical trials that can make the data applicable to the whole paediatric population and that can be transposed into law by the drug regulation authorities. The use of first-generation H1-antihistamines (es. hydroxyzine) is not recommended [165, 171, 172]. They are poorly selective against H1 receptor and can easily cross the blood-brain barrier. Consequently, they more frequently determine adverse event than second-generation antihistamines, including sedation, dry mouth, headache, blurred vision, glaucoma, urinary retention [162].

**Table 5:** Second-generation anti-H1 anti-histamine for children

Second-generation H1-antihistamines	Pharmaceutical form	Age and/or weight	Dose
Cetirizine	Drops 10 mg/ml (1 drop = 0,5 mg) Oral solution 1 mg/ml Tablets 10 mg	2–6 years	2.5 mg b.i.d.
		6–12 years	5 mg b.i.d.
		12–18 years	10 mg q.d.
Loratadine	Syrup 1 mg/ml Tablets 10 mg	2–12 years (< 30 kg)	5 mg q.d.
		2–12 years (> 30 kg)	10 mg q.d.
		12–18 years	10 mg q.d.
Fexofenadine	Tablets 120 mg Tablets 180 mg (Tablets/syrup 30 mg in US/UK)	12–18 years (US> 2 years, UK > 6 years)	120–180 mg q.d. 30 mg q.d.
Levocetirizine	Drops 5 mg/ml (1 drop = 0,25 mg) Tablets 5 mg	2–6 years (in US > 6 months)	1.25 mg q.d.
		*>* 6 years	5 mg q.d.
Desloratadine	Oral solution 0,5 mg/ml Tablets 2.5 mg Tablets 5 mg	1–5 years	1.25 mg q.d.
		6–11 years	2.5 mg q.d.
		> 12 years	5 mg q.d.
Acrivastine	Syrup 8 mg/10 ml Tablets 8 mg	> 12 years	8 mg t.i.d.
Rupatadine	Oral solution 1 mg/ml Tablets 10 mg	2–11 years (< 25 kg)	2.5 mg q.d.
		2–11 years (> 25 Kg)	5 mg q.d.
		> 12 years old	10 mg q.d.
Bilastine	Syrup 2.5 mg/ml Tablets 10 mg Tablets 20 mg	> 6 years (> 20 kg)	10 mg q.d.
		> 12 years	20 mg q.d.
Ebastine	Tablets 10 mg Tablets 20 mg (Syrup 5 mg/ml)	> 12 years (> 2 aa in Europe)	10–20 mg q.d. (0.2 mg/kg q.d.)

*Question 21. Is there any evidence of greater efficacy of a H1-antihistamine compared to the others? In case of failure of H1-antihistamine at standard dosing, should a different H1-antihistamine be used?*

*Answer: There is no evidence that any H1-antihistamine is more effective than the others in the treatment of CU, therefore no specific H1-antihistamine is recommended as a first option. (Level of evidence I. Strength of recommendation D.)*

The efficacy of the available H1-antihistamines at standard doses has been evaluated in a recent systematic review [162]. Desloratadine exhibited superior efficacy than placebo in inducing complete remission in medium-term (5 mg q.d./2 weeks-3 months) and short-term (20 mg q.d./2 weeks) therapies, although no difference was observed between 5 mg q.d. and 10 mg q.d. or short-term treatments. Comparisons between loratadine (10 mg q.d.) vs placebo and vs cetirizine (10 mg q.d.) in short- and medium-term therapies did not show significant differences in terms of “good or excellent response” or complete remission of CU. No significant differences were found between loratadine (10 mg q.d.) vs desloratadine (5 mg q.d.) in medium-term therapy efficacy. Loratadine (10 mg q.d.) and hydroxyzine (25 mg q.d.) were found to be effective and comparable to each other in inducing complete remission in short-term treatments. There was no difference between loratadine (10 mg q.d.) and mizolastine (10 mg q.d.) in terms of complete remission of symptoms and improvement of the quality of life ≥50% [162]. Levocetirizine was effective at a dose of 5 mg/day in medium-term therapies, but not in short-term ones, while a higher dose (20 mg q.d.) proved to be effective in short-term therapy. In comparative studies, levocetirizine (5–20 mg q.d.) was more effective than desloratadine (5–20 mg q.d.) [162]. Cetirizine has been shown to determine the remission of CU in more patients then fexofenadine [162]. The authors [162] concluded that none of second generation H1-antihistamines was more effective than the others in the control of CU symptoms, although the quality of evidence was heterogeneous.

Adverse events of H1-antihistamine have some inter-individual variability, some subjects “tolerate” an antihistamine better than another [162, 173]. In a placebo-controlled comparative study, there were no significant differences in drug withdrawal rates due to adverse events between the active group (cetirizine 10 mg q.d. and 20 mg q.d., desloratadine 5 mg q.d., hydroxyzine 25 mg q.d.) and placebo [162].

Rupatadine at standard doses (10 mg q.d.) has a good tolerability and safety profile in children aged 2–11 years. In a double-blind, randomized, placebo-controlled study carried out in children aged 2–11 years with CSU, no significant differences were found in reducing wheals between desloratadine and rupatadine, although rupatadine but not desloratadine was statistically superior to placebo in reduction of pruritus (− 57%). Children’s quality of life was statistically improved both in subjects treated with rupatadine and with desloratadine compared to placebo. The incidence of adverse events was equal to placebo in both active groups [158].

In a prospective, open, randomized study [168] in 100 patients aged 12–65 years old, levocetirizine was found to be more effective than rupatadine in CU patients, but both drugs caused mild sedation.

*Question 22. In case of failure of second-generation H1-antihistamine at standard dosing, what are the options? Should the dosage of H1-antistamines be increased? If there is no control of symptoms, should a different H1-antihistamine be prescribed?*

*Recommendation. In children over 12 years of age, if standard dosing of second-generation H1-antihistamine does not adequately control CU, after evaluation of the risk-benefit ratio, an increase in the daily dosage (by increasing frequency of administration) up to fourfold may be recommended (off-label) (Level of evidence I. Strength of recommendation B)*

*In children <12 years, although no study is available, increasing the dosage can be evaluated, considering that doubling daily doses of second-generation H1-antihistamines has been proved safe in large controlled studies (Level of Evidence I. Strength of Recommendation C). Although there is no evidence of relevant clinical difference among new H1 antagonists, in patients who do not respond, a course of treatment with a different molecule can be tried. (Level of Evidence VI. Strength of Recommendation C).*

Some authors have described the benefit of a higher dosage (up to fourfold) of second generation H1-antihistamines, in order to control symptoms without compromising the safety profile of these drugs [162, 174]. This approach is recommended by European guidelines [7] based on the assessment of the risk-benefit ratio [175] The efficacy of this approach has been shown in randomized controlled studies on adolescents aged> 12 years old and adults using up to fourfold higher dosage than standard one of cetirizine, fexofenadine, bilastine, ebastine, desloratadine in CU [163, 176] and desloratadine, rupatadine and bilastine in cold-induced urticaria [177, 178]., without significant increasing in side effects. Other studies have observed the efficacy of H1-antistamines at increased dosage [179–183]. The evidence about the use of H1-antihistamines at increased dosage over long term is not yet available.

*Question 23. When second-generation H1-antihistamines are not adequate to control CU, should a combination of second-generation H1-antistamine and first-generation H1-antihistamine or H2-antihistamine be given?*

*Recommendation. A combination of second-generation H1-antistamine and first-generation H1-antihistamine, or of H1-and H2-antagonists should not be given in CU. (Level of evidence I. Strength of recommendation D).*

Few studies have evaluated the combined use of different anti-H1 antihistamines at standard or increased dosage. A systematic review concludes that there is no evidence for recommending this option, although it is sometimes used in clinical practice [162]. In adults with CU, adding a first-generation H1-antihistamine (hydroxyzine) to a second generation H1-antihistamine (levocetirizine) is not more effective than levocetirizine alone [184]. A systematic review, including studies in adults, pointed out that the evidence about the efficacy of H2-antagonists for the treatment of CU is weak and unreliable [185].

*Question 24. When second-generation H1-antihistamine do not adequately control CU, could other treatments be recommended in children?*

**Fig. 2:**
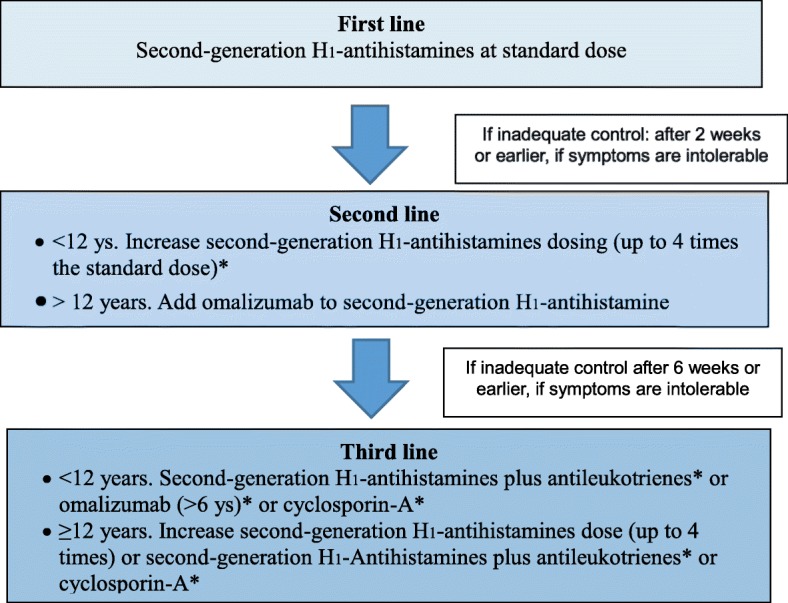
Treatment of CU in children. *off-label

Several treatments have been proposed for use as second and third line therapy in antihistamines-refractory patients. In children, clinical trials on these therapies are lacking or of low quality, except for omalizumab. Therefore, the strength of recommendation is weak, except for omalizumab because of little or no evidence of efficacy, high costs and frequent poor tolerability. When these treatments are started, antihistamines and other drugs that were helpful to the patient should be continued (Fig. 2).

### Omalizumab

*Recommendation. In patients 12 years of age and older with CSU, omalizumab should be added to second-generation H1-antistamines as a second-line therapy when second-generation H1-antistamines alone do not give adequate relief. (Level of evidence I. Strength of recommendation A)* (Figure 2*)*

Omalizumab, a monoclonal antibody against IgE, is approved for the treatment of children with CSU 12 years of age and older when CSU is not controlled by H1-antihistamine. Studies on omalizumab for the treatment of CSU were mainly performed on adult subjects, and in some cases, paediatric patients (> 12 years old) were also included. Omalizumab achieved statically significant reduction of clinical score and it was safe. Three randomized controlled trials [186–188] including paediatric patients are available. Ninety patients (5 aged < 18 years) with UAS7 >  12 where analysed in a prospective, randomized, quadruple-blind, placebo-controlled, dose-ranging study. They were randomized to receive placebo or omalizumab every 4 weeks in 3 different dose injections (75 mg, 300 mg, 600 mg). Both the 300-mg omalizumab group and the 600-mg omalizumab group showed greater improvement than the placebo group in UAS7 (13.0 e 7.7 points respectively) [186]. The multicentre phase III ASTERIA II study, randomly assigned 323 patients (10 aged < 18 years) with CSU resistant to standard H1-antihistamine therapy and UAS7 > = 16 to receive omalizumab at doses of 75 mg, 150 mg, or 300 mg or placebo, during a period of 12 weeks. The authors found a significant reduction in the average itch-severity-scores (ISS) in the group receiving doses of 150 and 300 mg (primary efficacy outcome), and also a reduction in UAS7, number of lesions, DLQI (Dermatology Life Quality Index), number of patients with UAS7 < 6 and proportion of patients with MID (minimally important difference) response in weekly itch-severity scores at 12 weeks (secondary outcome). Adverse events observed showed no significant differences in the different groups, although the rate was higher in the group treated with 300 mg [187]. The multicentre phase III ASTERIA I trial, a randomized, double-blind, placebo-controlled study, evaluated the efficacy and safety of omalizumab in 319 patients (18 of whom < 18 years). Patients were randomized to receive omalizumab 75 mg, 150 mg, or 300 mg or placebo for 24 weeks. The treatment groups (75 mg, 150 mg and 300 mg) had significant improvement in secondary outcomes and maintenance of efficacy at 24 weeks compared to placebo, without significant adverse events [188]. The three trials [186–188] had a low risk of bias in randomization, masking, blindness, dropouts during treatment or follow-up, coherence in reporting. Potential biases were represented by the sponsorship of the studies and by the limited number of paediatric patients.

Two other randomized trials have shown the efficacy and safety of omalizumab in adult patients [189, 190]. The response was often observed within a week after the first dose and there are slow responders after 3–5 months. Patients usually relapse after a few months when omalizumab is stopped.

A prospective open-label (real-life) study evaluated the efficacy of 150 mg of omalizumab, at intervals ranging from 15 days to 7 weeks, in 68 patients with severe refractory urticaria. 78% achieved complete remission during omalizumab therapy (UAS-7 0) [191]. Another study treated 47 CU patients aged from 16 to 74 years with Omalizumab at a dose of 150 mg/month or 300 mg/month. 84% of patients treated with the highest dose achieved clinical remission. Of the 20 patients who started the treatment with 150 mg of omalizumab, 12 (60%) had a complete response. In 6 of the partial responders, a higher dose of 300 mg was used: 4 of them (66.7%) had a complete resolution of symptoms with 300 mg but 2 still have symptoms [192].

Despite the lack of studies in children, the trials published up to date showed efficacy and tolerability of omalizumab. The small number of adolescents who were enrolled in the phase 3 trials [186–188] is a limitation. Whether the baseline clinical findings reported [186–188] can be generalized for this patient population is unknown. This, in combination with the small number of real-world studies, highlights the need for larger studies focusing on efficacy of omalizumab in the subgroup of adolescents with CSU. Case reports of efficacy of omalizumab in children less than 12 years of age have been reported [193, 194]. A limitation of omalizumab is the cost that may be unaffordable in many circumstances. Regarding CIU, there are sparse reports of efficacy of omalizumab in children with cold urticaria [195–197] and solar urticaria [198].

### Cyclosporin-A

*Recommendation. The use of ciclosporin-A can be considered when the combination of second-generation H1-antistamines and omalizumab is not enough to control CSU or patients have not access to omalizumab. Its use is limited by possible side effects (Level of evidence V. Strength of recommendation C).*

The efficacy of cyclosporin A has been shown in some children with CSU, that was not controlled by high-dose antihistamine [199] or the combination of antihistamine and prednisone [200].

In a prospective, open-label study in 30 patients aged over 18 years, a 5-month cyclosporin-A course has shown good efficacy, 87% of patients was symptom-free after one year of follow-up but there was also a significant number (7/30) of dropouts due to adverse events and failure of low-dose therapy [201]. In adults, cyclosporine A is less effective than omalizumab [202]. Prescription of Ciclosporin A is off-label.

### Systemic corticosteroids

*Recommendation. A short course (up to 10 days) of systemic glucocorticoids can be used during* severe CU *exacerbations. (Level of evidence VI. Strength of recommendation B.)*

*Long-term treatment with systemic glucocorticoids should be avoided because of the risk of significant side effects (Level of evidence VI: Strength of recommendation E).*

Oral corticosteroids should be used as a rescue therapy in severe CU exacerbations. There are no controlled studies on the use of corticosteroids in patients with CU, although they are useful in clinical practice to control symptoms [203]. A retrospective cohort study found that the use of systemic corticosteroids in CU increased risk of corticosteroid-related adverse events and health care costs compared to patients not treated with steroids [204]. Corticosteroids should be given for short periods (3–10 days) given the unacceptable adverse events of their long-term use [7].

### Montelukast

*Recommendation. Montelukast in children with CU may be added to a second generation H1-antistamine if they do not control symptoms at standard dose (Level of evidence VI. Strength of Recommendation C).*

There are no paediatric studies on montelukast in CU. In adults, some randomized trials on montelukast alone in CSU did not improve symptoms better than H1-antistamine, while there was weak evidence of efficacy when it is added to H1-antistamine [205–207]. The choice of this drug can also be justified by its excellent safety profile.

### Other therapies

*Recommendation. There is insufficient data to evaluate indication of other treatments in paediatric CU (Level of evidence VI. Strength of Recommendation D).*

Methotrexate is a drug of uncertain efficacy in CSU, considering the scarcity of studies concerning its efficacy and tolerability [208, 209]. Moreover, there are no data in children. There is no evidence of efficacy in children for the following drugs: sulfasalazine, interferon, plasmapheresis, phototherapy, immunoglobulin ev, danazol, warfarin, ac. tranexamic, hydroxychloroquine, rituximab, heparin, anakinra, anti-TNF alpha, colchicine, miltefosine, mirtazapine, camostat mesylate, mycophenolate mofetil [7, 210].

A systematic review analysed the efficacy of allergen-specific immunotherapy in CU, including 2 very low-quality paediatric studies that would demonstrate significant efficacy in improving urticaria symptoms [211]. Allergen-specific immunotherapy in CU and in atopic dermatitis [212] is supported by preliminary evidence of effectiveness as opposed to respiratory allergies [213]. A controlled study analysed the use of atorvastatin in combination with an antihistamine [214], another randomized the use of levothyroxine in euthyroid patients with positive blood antibodies [215]. In a randomized parallel single blind trial, performed on 88 adult patients, no significant difference was found in the improvement of CSU after the injection of autologous whole blood or autologous serum or placebo after 6 weeks of treatment [216]. A randomized study on 24 patients with CSU, aged between 14 and 58 years old, who were treated with PUVA or with NB-UVB and evaluated at 20 weeks, did not find any significant differences in the efficacy of both therapies [217]. An open study is available on the use of vitamin D in CSU in 57 patients aged 14 to 75 years old, with vitamin D values below 30 mcg/L. They were treated with 300.000 IU/month for 3 months and a significant improvement of UAS4 and Chronic Urticaria Quality of Life Questionnaire (CU-Q2oL) was found [218]. Therefore, vitamin D supplementation can be helpful in patients with demonstrated vitamin D deficiency. The efficacy of a Peony derivative in combination or not with cetirizine was examined in a randomized study in patients aged between 16 and 65, with significant results although not standardized [219]. The effects of herbs were described in a randomized trial of patients of unknown age [220].

Question 26. Does CU have an impact on patient's quality of life, and which is the burden of the disease on psychological aspects? How should psychological distress be addressed?

Recommendation

• *CU affects children's quality of life and their families. The most effective instrument to evaluate quality of life is the care relationship. When necessary, however, the CU-Q2oL can be useful. (Level of evidence V. Strength of recommendation B.)*

• A multidimensional strategy which includes psychoeducational and behavioural interventions, would be appropriate for all patients with CU. Regular monitoring of the patient's emotional state through periodic (every six-months) psychological counselling can reduce maladaptive strategies, prevent or timely identify the onset of significant psychological problems, and intervene timely (Level of evidence VI. Strength of Recommendation B).

• *It is recommended to investigate the presence of anxiety, depressive symptoms, isolation or stressful events in patients with CU, as well as to evaluate any signs of psychological or relational distress in patient's parents and/or siblings (Level of evidence VI. Strength of recommendation B)*

• *It is recommended that the multidisciplinary team has a "psychological approach", oriented to empathic listening, availability, clarity, and using a shared language. (Trial Level VI Strength of Recommendation B).*

• *It is recommended that referral of the patient to a psychological consultation happens in a valid team/patient/family relationship. Is should not be a delegation, but a common route (Level of evidence VI. Strength of recommendation B).*

• *Type of the psychoeducational approach (individual or group) varies according to the doctor's availability and family’s needs and availability (Level of evidence VI Strength of recommendation B).*

### Psychological impact and quality of life

Several studies agree that CU is a disabling skin disease with a very significant impact on patient’s psychological state and quality of life. In a mixed population of children and adults, higher levels of anxiety and depression were found in patients with an uncertain diagnosis [221]. The most used tool for assessing children’s quality of life is Children’s Dermatology Life Quality Index (CDLQ) [222]. CU-Q2oL has been recommended [7]. Unfortunately, all the studies on psychological aspects and impact on the quality of life were carried out on adult patients and there is very little on children. A paediatric disease is an event that may influence the parent-child relationship, becoming very important and central in family life. Parents can experience feelings of guilt and frustration, financial strain, inadequacy that can be “silently” transmitted to the child, who can perceive themselves as very ill, and create a personal identity that revolves around the disease. In this context, the characteristics of the family, its resources, the social context are very important to avoid the onset of possible experiences of diversity, limitation, fragility and non-amiability in the child. Several studies reported that, in children with CU, discomfort caused by itching, aesthetic aspect and unpredictability of manifestations can cause, in the children, concern about their health and some internalizing symptoms, anxiety, a higher risk of depression, in a circular reaction in which it is difficult to identify causes and effects [126, 223, 224] .

Children with CU mainly complain about itching and pain with emotional, behavioural and relational impairment and a negative impact on quality of life [5, 222].

Perception of pain and pruritus can be influenced by an individual emotional component, due to patient characteristics, stressful events, and family attitude towards the disease. Studies have reported that parents of patients with CU report feelings of fatigue, despair and sleep disorders, as well as a constant time commitment to therapy and check-ups.

### Monitoring and approach of emotional/psychological distress

Some studies have affirmed that, since children with CU have high psychiatric morbidity, their psychological status should be screened by clinicians. A regular six-monthly monitoring allows early detection of signs of discomfort such as tension, anxiety, depressive feelings, social isolation, somatic complaints, sleep and eating disorders, bad school performance. A psychiatric evaluation of all family members is necessary to investigate any personal and/or couple problems, feelings of inadequacy, guilt and inability to give attention to other children, sibling rivalry because of attention focused on the child with disease.

In order to be effective, psychological consultation must take place within a valid and trust-based relationship between clinicians, patient and his family. The referral must be “protected and accompanied” because the emotional area is considered an integral part of the treatment process. It is desirable that the first psychological consultation takes place in the presence of the attending specialist, sharing information about the necessary therapeutic interventions, that must be well explained to the family.

Therapeutic programs must be integrated and beneficial, they can be individualized or group-based (paediatrician-psychologist plus any other specialists such as dermatologist, allergist or immunologist) [225]. The group psychoeducational intervention allows child and his family to confront with other patients, reducing the feeling of isolation, loneliness and diversity. The psychoeducational approach presupposes a holistic vision, based on collaboration, skills increasing, coping and mobilization of the resources of the patient and his parents.

## Data Availability

Not applicable.

## Abbreviations

AE: Angioedema
APST: Autologous plasma skin test
ASST: Autologous serum skin test
AU: Acute urticaria
BAT: Basophil activation test
BHRA: Basophil histamine release assay
CIU: Chronic inducible urticaria
CSU: Chronic spontaneous urticaria
CU: Chronic urticaria
CU-Q2oL: Chronic urticaria quality of life questionnaire
NSAIDs: Non steroidal anti-inflammatory drugs
OFC: Oral food challenge
UAS: Urticaria activity score
VU: Vasculitic urticaria

## References

[1] Levy Y, Segal N, Weintrob N, Danon YL (2003). Chronic urticaria: association with thyroid autoimmunity. Arch Dis Child.

[2] Caminiti L, Passalacqua G, Magazzù G, Comisi F, Vita D, Barberio G (2005). Chronic urticaria and associated coeliac disease in children: a case–control study. Pediatr Allergy Immunol.

[3] Hoffman WH, Helman SW, Sekul E, Carroll JE, Vega RA (2003). Lambert–Eaton myasthenic syndrome in a child with an autoimmune phenotype. Am J Med Genet A.

[4] Church MK, Weller K, Stock P, Maurer M (2011). Chronic spontaneous urticaria in children: itching for insight. Pediatr Allergy Immunol.

[5] Lewis-Jones MS (2006). A comparative study of impairment of quality of life in children with skin disease and children with other chronic childhood diseases. Br J Dermatol.

[6] Powell RJ, Leech SC, Till S, Huber PA, Nasser SM, Clark AT, British Society for Allergy and Clinical Immunology (2015). BSACI guideline for the management of chronic urticaria and angioedema. Clin Exp Allergy.

[7] Zuberbier T, Aberer W, Asero R, Abdul Latiff AH, Baker D, Ballmer-Weber B (2018). The EAACI/GA2LEN/EDF/WAO guideline for the definition, classification, diagnosis, and management of urticaria. Allergy..

[8] Bernstein JA, Lang DM, Khan DA, Craig T, Dreyfus D, Hsieh F (2014). The diagnosis and management of acute and chronic urticaria: 2014 update. J Allergy Clin Immunol.

[9] Limpongsanurak W, Tuchinda P, Chularojanamontri L, Chanyachailert P, Korkij W, Chunharas A (2016). Clinical practice guideline for diagnosis and management of urticaria. Asian Pac J Allergy Immunol.

[10] Katelaris CH, Smith W, Choi J, Frith K, Lau WY, Nolan R, et al. ASCIA chronic spontaneous Urticaria (CSU) guidelines. 2015. https://www.allergy.org.au/hp/papers/chronic-spontaneous-urticaria-csu-guidelines.

[11] Beck LA, Bernstein JA, Maurer M (2017). A review of international recommendations for the diagnosis and management of chronic urticaria. Acta Derm Venereol.

[12] Caffarelli C, Cardinale F, Paravati F (2009). Linea Guida italiane sull’orticaria cronica in età pediatrica. Minerva Pediatr.

[13] Istituto Superiore di Sanità. Istituto Superiore di Sanità. https://www.iss.it. Accessed 20 Mar 2019.

[14] Grilli R, Magrini N, Penna A, Mura G, Liberati A (2000). Practice guidelines developed by specialty societies: the need for a critical appraisal. Lancet.

[15] Shea BJ, Hamel C, Wells GA, Bouter LM, Kristjansson E, Grimshaw J (2009). AMSTAR is a reliable and valid measurement tool to assess the methodological quality of systematic reviews. J Clin Epidemiol.

[16] Shea BJ, Reeves BC, Wells G, Thuku M, Hamel C, Moran J (2017). AMSTAR 2: a critical appraisal tool for systematic reviews that include randomised or non-randomised studies of healthcare interventions, or both. Bmj.

[17] Deeks JJ, JPT H, Altman DG, Green S. Cochrane handbook for systematic reviews of interventions version 5.1. 0 (updated March 2011). Cochrane Collab. 2011.

[18] Wells GA, Shea B, O'Connell D, Peterson J, Welch V, Losos M (2009). The Newcastle-Ottawa scale (NOS) for assessing the quality of nonrandomised studies in meta-analyses.

[19] Whiting PF, Rutjes AW, Westwood ME, Mallett S, Deeks JJ, Reitsma JB (2011). QUADAS-2: a revised tool for the quality assessment of diagnostic accuracy studies. Ann Intern Med.

[20] Prognosis | Users’ Guides to the Medical Literature: A Manual for Evidence-Based Clinical Practice, 3rd ed | JAMA evidence | McGraw-Hill Medical. http://jamaevidence.mhmedical.com/content.aspx?bookid=847&sectionid=69031497.

[21] Magen E, Zueva E, Mishal J, Schlesinger M (2016). The clinical and laboratory characteristics of acute spontaneous urticaria and its progression to chronic spontaneous urticaria. Allergy Asthma Proc..

[22] Nettis E, Dambra P, D'Oronzio L, Cavallo E, Loria MP, Fanelli M (2002). Reactivity to autologous serum skin test and clinical features in chronic idiopathic urticaria. Clin Exp Dermatol.

[23] Song Z, Zhai Z, Zhong H, Zhou Z, Chen WC, Hao F (2013). Evaluation of autologous serum skin test and skin prick test reactivity to house dust mite in patients with chronic spontaneous urticaria. PLoS One.

[24] Bernstein JA, Cremonesi P, Hoffmann TK, Hollingsworth J (2017). Angioedema in the emergency department: a practical guide to differential diagnosis and management. Int J Emerg Med.

[25] Magerl M, Altrichter S, Borzova E, Gimenez-Arnau A, Grattan CHE, Lawlor F (2016). The definition, diagnostic testing, and management of chronic inducible urticarias–the EAACI/GA2LEN/EDF/UNEV consensus recommendations 2016 update and revision. Allergy.

[26] Ying S, Kikuchi Y, Meng Q, Kay AB, Kaplan AP (2002). TH1/TH2 cytokines and inflammatory cells in skin biopsy specimens from patients with chronic idiopathic urticaria: comparison with the allergen-induced late-phase cutaneous reaction. J Allergy Clin Immunol.

[27] Sabroe RA, Seed PT, Francis DM, Barr RM, Black AK, Greaves MW (1999). Chronic idiopathic urticaria: comparison of the clinical features of patients with and without anti-FcϵRI or anti-IgE autoantibodies. J Am Acad Dermatol.

[28] Jirapongsananuruk O, Pongpreuksa S, Sangacharoenkit P, Visitsunthorn N, Vichyanond P (2010). Identification of the etiologies of chronic urticaria in children: a prospective study of 94 patients. Pediatr Allergy Immunol.

[29] Fukunaga A, Bito T, Tsuru K, Oohashi A, Yu X, Ichihashi M, Nishigori C, Horikawa T (2005). Responsiveness to autologous sweat and serum in cholinergic urticaria classifies its clinical subtypes. J Allergy Clin Immunol.

[30] Kim JE, Jung KH, Cho HH, Kang H, Park YM, Park HJ, Lee JY (2015). The significance of hypersensitivity to autologous sweat and serum in cholinergic urticaria: cholinergic urticaria may have different subtypes. Int J Dermatol.

[31] Zuberbier T, Balke M, Worm M, Edenharter G, Maurer M (2010). Epidemiology of urticaria: a representative cross-sectional population survey. Clin Exp Dermatol.

[32] Lee SJ, Ha EK, Jee HM, Lee KS, Lee SW, Kim MA (2017). Prevalence and risk factors of urticaria with a focus on chronic urticaria in children. Allergy Asthma Immunol Res..

[33] Cantarutti A, Donù D, Visentin F, Borgia E, Scamarcia A, Cantarrutti L (2015). Epidemiology of frequently occurring skin diseases in Italian children from 2006 to 2012: a retrospective. Population-Based Study Pediatr Dermatol.

[34] Broder MS, Raimundo K, Antonova E, Chang E (2015). Resource use and costs in an insured population of patients with chronic idiopathic/spontaneous urticaria. Am J Clin Dermatol.

[35] Brüske I, Standl M, Weidinger S, Klümper C, Hoffmann B, Schaaf B (2014). Epidemiology of urticaria in infants and young children in Germany–results from the German LISAplus and GINIplus birth cohort studies. Pediatr Allergy Immunol.

[36] Harris A, Twarog FJ, Geha RS (1983). Chronic urticaria in childhood: natural course and etiology. Ann Allergy.

[37] Du Toit G, Prescott R, Lawrence P, Johar A, Brown G, Weinberg EG (2006). Autoantibodies to the high-affinity IgE receptor in children with chronic urticaria. Ann Allergy Asthma Immunol.

[38] Sahiner UM, Civelek E, Tuncer A, Yavuz ST, Karabulut E, Sackesen C, Sekerel E (2011). Chronic urticaria: etiology and natural course in children. Int Arch Allergy Immunol.

[39] Chansakulporn S, Pongpreuksa S, Sangacharoenkit P, Pacharn P, Visitsunthorn N, Vichyanond P, Jirapongsananuruk O (2014). The natural history of chronic urticaria in childhood: a prospective study. J Am Acad Dermatol.

[40] Eser I, Yologlu N, Baydemir C, Aydogan M (2016). The predictive factors for remission of chronic spontaneous urticaria in childhood: outcome from a prospective study. Allergol Immunopathol (Madr).

[41] Arik Yilmaz E, Karaatmaca B, Cetinkaya PG, Soyer O, Sekerel BE, Sahiner UM (2017). The persistence of chronic spontaneous urticaria in childhood is associated with the urticaria activity score. Allergy Asthma Proc.

[42] Netchiporouk E, Sasseville D, Moreau L, Habel Y, Rahme E, Ben-Shoshan M (2017). Evaluating comorbidities, natural history, and predictors of early resolution in a cohort of children with chronic urticaria. JAMA Dermatol.

[43] Quaranta JH, Rohr AS, Rachelefsky GS, Siegel SC, Katz RM, Spector SL, Mickey MR (1989). The natural history and response to therapy of chronic urticaria and angioedema. Ann Allergy.

[44] Kozel MM, Mekkes JR, Bossuyt PM, Bos JD (2001). Natural course of physical and chronic urticaria and angioedema in 220 patients. J Am Acad Dermatol.

[45] Toubi E, Kessel A, Avshovich N, Bamberger E, Sabo E, Nusem D, Panasoff J (2004). Clinical and laboratory parameters in predicting chronic urticaria duration: a prospective study of 139 patients. Allergy..

[46] Hiragun M, Hiragun T, Mihara S, Akita T, Tanaka J, Hide M (2013). Prognosis of chronic spontaneous urticaria in 117 patients not controlled by a standard dose of antihistamine. Allergy..

[47] Khakoo G, Sofianou-Katsoulis A, Perkin MR, Lack G (2008). Clinical features and natural history of physical urticaria in children. Pediatr Allergy Immunol.

[48] Kim HJ, Lee MG (2017). Cholinergic urticaria: more than a simple inducible urticaria. Australas J Dermatol.

[49] Kim JE, Eun YS, Park YM, Park HJ, Yu DS, Kang H (2014). Clinical characteristics of cholinergic urticaria in Korea. Ann Dermatol.

[50] Seol JE, Kim DH, Park SH, Kang JN, Sung HS, Kim H (2017). Aquagenic Urticaria diagnosed by the water provocation test and the results of histopathologic examination. Ann Dermatol.

[51] Rothbaum R, McGee JS (2016). Aquagenic urticaria: diagnostic and management challenges. J Asthma Allergy.

[52] Alangari AA, Twarog FJ, Shih MC, Schneider LC (2004). Clinical features and anaphylaxis in children with cold urticaria. Pediatrics.

[53] Jain SV, Mullins RJ (2016). Cold urticaria: a 20-year follow-up study. J Eur Acad Dermatol Venereol.

[54] Beattie PE, Dawe RS, Ibbotson SH, Ferguson J (2003). Characteristics and prognosis of idiopathic solar urticaria: a cohort of 87 cases. Arch Dermatol.

[55] Caffarelli C, Cuomo B, Cardinale F, Barberi S, Povesi Dascola C, Agostinis F (2013). Aetiological factors associated with chronic urticaria in children: a systematic review. Acta Derm Venereol.

[56] Azkur D, Civelek E, Toyran M (2016). Clinical and etiologic evaluation of the children with chronic urticaria. Allergy Asthma Proc..

[57] Yilmaz EA, Karaatmaca B, Sackesen C, Sahiner UM, Cavkaytar O, Sekerel BE, Soyer O (2016). Parasitic infections in children with chronic spontaneous urticaria. Int Arch Allergy Immunol.

[58] Brunetti L, Francavilla R, Miniello VL, Platzer MH, Rizzi D, Lospalluti ML (2004). High prevalence of autoimmune urticaria in children with chronic urticaria. J Allergy Clin Immunol.

[59] Volonakis M, Katsarou-Katsari A, Stratigos J (1992). Etiologic factors in childhood chronic urticaria. Ann Allergy.

[60] Sackesen C, Sekerel BE, Orhan F, Kocabas CN, Tuncer A, Adalioglu G (2004). The etiology of different forms of urticaria in childhood. Pediatr Dermatol.

[61] Dice JP (2004). Physical urticaria. Immunol Allergy Clin..

[62] Magerl M, Borzova E, Giménez-Arnau A, Grattan CE, Lawlor F, Mathelier-Fusade P (2009). The definition and diagnostic testing of physical and cholinergic urticarias-EAACI/GA2LEN/EDF/UNEV consensus panel recommendations. Allergy.

[63] Murphy GM, Zollman PE, Greaves MW, Winkelmann RK (1987). Symptomatic dermographism (factitious urticaria)--passive transfer experiments from human to monkey. Br J Dermatol.

[64] Abajian M, Mlynek A, Maurer M (2012). Physical urticaria. Curr Allergy Asthma Rep.

[65] Abajian M, Schoepke N, Altrichter S, Zuberbier T, Maurer M (2014). Physical urticarias and cholinergic urticaria. Immunol Allergy Clin.

[66] Sánchez-Borges M, González-Aveledo L, Caballero-Fonseca F, Capriles-Hulett A (2017). Review of physical urticarias and testing methods. Curr Allergy Asthma Rep.

[67] Pezzolo E, Peroni A, Gisondi P, Girolomoni G (2016). Heat urticaria: a revision of published cases with an update on classification and management. Br J Dermatol.

[68] Bird JA, Burks W (2010). Peanut allergy saves a patient with cold-induced hypotension and urticaria. J Pediatr.

[69] Kogame T, Uetsu N, Nguyen CTH, Kawada A, Okamoto H (2017). Solar urticaria with an augmentation spectrum in a child. J Dermatol.

[70] Wedi B, Raap U, Wieczorek D, Kapp A (2009). Urticaria and infections. Allergy Asthma Clin. Immunol..

[71] Kolkhir P, Balakirski G, Merk HF, Olisova O, Maurer M (2016). Chronic spontaneous urticaria and internal parasites–a systematic review. Allergy..

[72] Tan RJ, Sun HQ, Zhang W, Yuan HM, Li B, Yan HT (2016). A 21–35 kDa mixed protein component from helicobacter pylori activates mast cells effectively in chronic spontaneous urticaria. Helicobacter.

[73] Shakouri A, Compalati E, Lang DM, Khan DA (2010). Effectiveness of helicobacter pylori eradication in chronic urticaria: evidence-based analysis using the grading of recommendations assessment, development, and evaluation system. Curr Opin Allergy Clin Immunol.

[74] Curth HM, Dinter J, Nigemeier K, Kütting F, Hunzelmann N, Steffen HM (2015). Effects of helicobacter pylori eradication in chronic spontaneous urticaria: results from a retrospective cohort study. Am J Clin Dermatol.

[75] Federman DG, Kirsner RS, Moriarty JP, Concato J (2003). The effect of antibiotic therapy for patients infected with helicobacter pylori who have chronic urticaria. J Am Acad Dermatol.

[76] Minciullo PL, Cascio A, Barberi G, Gangemi S (2014). Urticaria and bacterial infections. Allergy Asthma Proc..

[77] Marseglia GL, Marseglia A, Licari A, Castellazzi AM, Ciprandi G (2007). Chronic urticaria caused by Hymenolepis nana in an adopted girl. Allergy..

[78] Casero RD, Mongi F, Sánchez A, Ramírez JD (2015). Blastocystis and urticaria: examination of subtypes and morphotypes in an unusual clinical manifestation. Acta Trop.

[79] López-Sáez MP, Zubeldia JM, Caloto M, Olalde S, Pelta R, Rubio M, Baeza ML (2003). Is Anisakis simplex responsible for chronic urticaria?. Allergy Asthma Proc..

[80] Ventura MT, Napolitano S, Menga R, Cecere R, Asero R (2013). Anisakis simplex hypersensitivity is associated with chronic urticaria in endemic areas. Int Arch Allergy Immunol.

[81] Imbalzano E, Casciaro M, Quartuccio S, Minciullo PL, Cascio A, Calapai G, Gangemi S (2016). Association between urticaria and virus infections: a systematic review. Allergy Asthma Proc..

[82] Dreyfus DH (2016). Serological evidence that activation of ubiquitous human herpesvirus-6 (HHV-6) plays a role in chronic idiopathic/spontaneous urticaria (CIU). Clin Exp Immunol.

[83] Shalom G., Magen E., Dreiher J., Freud T., Bogen B., Comaneshter D., Vardy D.A., Khoury R., Agmon-Levin N., Cohen A.D. (2017). Chronic urticaria and atopic disorders: a cross-sectional study of 11 271 patients. British Journal of Dermatology.

[84] Magen E, Mishal J, Menachem S (2011). Impact of contact sensitization in chronic spontaneous urticaria. Am J Med Sci.

[85] Hession MT, Scheinman PL (2012). The role of contact allergens in chronic idiopathic urticaria. Dermatitis.

[86] Chen H, Liu G, Huang N, Li W, Dong X, Zhu R (2016). Incidence of allergic contact sensitization in central Chinese subjects with chronic urticaria. An Bras Dermatol.

[87] Kessel A, Helou W, Bamberger E, Sabo E, Nusem D, Panassof J, Toubi E (2010). Elevated serum total IgE--a potential marker for severe chronic urticaria. Int Arch Allergy Immunol.

[88] Chang KL, Yang Y-H, Yu H-H, Lee J-H, Wang L-C, Chiang B-L (2013). Analysis of serum total IgE, specific IgE and eosinophils in children with acute and chronic urticaria. J Microbiol Immunol Infect.

[89] Hsu M-L, Li L-F (2012). Prevalence of food avoidance and food allergy in Chinese patients with chronic urticaria. Br J Dermatol.

[90] Asero R (2011). Hypersensitivity to lipid transfer protein is frequently associated with chronic urticaria. Eur Ann Allergy Clin Immunol.

[91] Asero R (2013). Chronic urticaria caused by allergy to peach lipid transfer protein. J Investig Allergol Clin Immunol.

[92] Kowalski ML, Woessner K, Sanak M (2015). Approaches to the diagnosis and management of patients with a history of nonsteroidal anti-inflammatory drug–related urticaria and angioedema. J Allergy Clin Immunol.

[93] Cavkaytar O, Arik Yilmaz E, Buyuktiryaki B, Sekerel BE, Sackesen C, Soyer OU (2015). Challenge-proven aspirin hypersensitivity in children with chronic spontaneous urticaria. Allergy.

[94] Cousin M, Chiriac A, Molinari N, Demoly P, Caimmi D (2016). Phenotypical characterization of children with hypersensitivity reactions to NSAIDs. Pediatr Allergy Immunol.

[95] Ehlers I, Niggemann B, Binder C, Zuberbier T (1998). Role of nonallergic hypersensitivity reactions in children with chronic urticaria. Allergy.

[96] Rajan JP, Simon RA, Bosso JV (2014). Prevalence of sensitivity to food and drug additives in patients with chronic idiopathic urticaria. J Allergy Clin Immunol Pract.

[97] Magerl M, Pisarevskaja D, Scheufele R, Zuberbier T, Maurer M (2010). Effects of a pseudoallergen-free diet on chronic spontaneous urticaria: a prospective trial. Allergy.

[98] Wagner N, Dirk D, Peveling-Oberhag A, Reese I, Rady-Pizarro U, Mitzel H, Staubach P (2017). A popular myth - low-histamine diet improves chronic spontaneous urticaria - fact or fiction?. J Eur Acad Dermatol Venereol.

[99] Confino-Cohen R, Chodick G, Shalev V, Leshno M, Kimhi O, Goldberg A (2012). Chronic urticaria and autoimmunity: associations found in a large population study. J Allergy Clin Immunol.

[100] Kolkhir P, Church MK, Weller K, Metz M, Schmetzer O, Maurer M (2017). Autoimmune chronic spontaneous urticaria: what we know and what we do not know. J Allergy Clin Immunol.

[101] Greaves MW, Tan KT (2007). Chronic urticaria: recent advances. Clin Rev Allergy Immunol.

[102] Asero R, Tedeschi A, Riboldi P, Cugno M (2006). Plasma of patients with chronic urticaria shows signs of thrombin generation, and its intradermal injection causes wheal-and-flare reactions much more frequently than autologous serum. J Allergy Clin Immunol.

[103] Asero R, Tedeschi A, Marzano AV, Cugno M. Chronic spontaneous urticaria: immune system, blood coagulation, and more: Taylor & Francis; 2016. 10.1586/1744666X.2016.1127160 26630120

[104] Fagiolo U, Kricek F, Ruf C, Peserico A, Amadori A, Cancian M (2000). Effects of complement inactivation and IgG depletion on skin reactivity to autologous serum in chronic idiopathic urticaria. J Allergy Clin Immunol.

[105] Konstantinou GN, Asero R, Maurer M, Sabroe RA, Schmid-Grendelmeier P, Grattan CE (2009). EAACI/GA(2) LEN task force consensus report: the autologous serum skin test in urticaria. Allergy..

[106] Netchiporouk E, Moreau L, Rahme E, Maurer M, Lejtenyi D, Ben-Shoshan M (2016). Positive CD63 basophil activation tests are common in children with chronic spontaneous urticaria and linked to high disease activity. Int Arch Allergy Immunol.

[107] Kolkhir P, Metz M, Altrichter S, Maurer M (2017). Comorbidity of chronic spontaneous urticaria and autoimmune thyroid diseases: a systematic review. Allergy..

[108] Altrichter S, Peter HJ, Pisarevskaja D, Metz M, Martus P, Maurer M (2011). IgE mediated autoallergy against thyroid peroxidase--a novel pathomechanism of chronic spontaneous urticaria?. PLoS One.

[109] Pan X-F, Gu J-Q, Shan Z-Y. The prevalence of thyroid autoimmunity in patients with urticaria: a systematic review and meta-analysis: Springer; 2015. 10.1007/s12020-014-0367-y 25064381

[110] Fine LM, Bernstein JA (2016). Guideline of chronic urticaria beyond. Allergy Asthma Immunol Res.

[111] Ludvigsson JF, Lindelöf B, Rashtak S, Rubio-Tapia A, Murray JA (2013). Does urticaria risk increase in patients with celiac disease? A large population-based cohort study. Eur J Dermatol.

[112] Kolkhir P, Pogorelov D, Olisova O, Maurer M (2016). Comorbidity and pathogenic links of chronic spontaneous urticaria and systemic lupus erythematosus–a systematic review. Clin Exp Allergy.

[113] Marrouche N, Grattan C (2012). Childhood urticaria. Curr Opin Allergy Clin Immunol.

[114] Baek YS, Jeon J, Kim JH, Oh CH (2014). Severity of acute and chronic urticaria correlates with D-dimer level, but not C-reactive protein or total IgE. Clin Exp Dermatol.

[115] Takahagi S, Mihara S, Iwamoto K, Morioke S, Okabe T, Kameyoshi Y, Hide M (2010). Coagulation/fibrinolysis and inflammation markers are associated with disease activity in patients with chronic urticaria. Allergy..

[116] Asero R (2013). D-dimer: a biomarker for antihistamine-resistant chronic urticaria. J Allergy Clin Immunol.

[117] Nishimura K, Kuzume K, Kagata Y (2016). Evaluation of dysfunction in blood coagulation in children with urticaria. Allergy.

[118] Chu CY, Cho YT, Jiang JH, Lin EI, Tang CH (2017). Epidemiology and comorbidities of patients with chronic urticaria in Taiwan: a nationwide population-based study. J Dermatol Sci.

[119] Shalom G, Magen E, Babaev M, Horev A, Freud T, Ben Yakov G, Comaneshter D, Vardy DA, Cohen AD (2018). Chronic urticaria and irritable bowel syndrome: a cross-sectional study of 11 271 patients. Br J Dermatol.

[120] Chen YJ, Wu C-Y, Shen J-L, Chen T-T, Chang Y-T (2012). Cancer risk in patients with chronic urticaria: a population-based cohort study. Arch Dermatol.

[121] Shalom G, Magen E, Babaev M, Tiosano S, Vardy DA, Linder D (2018). Chronic urticaria and the metabolic syndrome: a cross-sectional community-based study of 11 261 patients. J Eur Acad Dermatol Venereol.

[122] Caffarelli C, Coscia A, Baldi F (2017). Characterization of irritable bowel syndrome and constipation in children with allergic diseases. Eur J Pediatr.

[123] Ben-Shoshan M, Blinderman I, Raz A (2013). Psychosocial factors and chronic spontaneous urticaria: a systematic review. Allergy.

[124] Theoharides TC, Stewart JM, Taracanova A, Conti P, Zouboulis CC (2016). Neuroendocrinology of the skin. Rev Endocr Metab Disord.

[125] Silvares MRC, Coelho KIR, Dalben I, Lastória JC, Abbade LPF (2017). Sociodemographic and clinical characteristics, causal factors and evolution of a group of patients with chronic urticaria-angioedema. Sao Paulo Med J.

[126] Hergüner S, Kılıç G, Karakoç S, Tamay Z, Tüzün Ü, Güler N (2011). Levels of depression, anxiety and behavioural problems and frequency of psychiatric disorders in children with chronic idiopathic urticaria. Br J Dermatol.

[127] Meni C, Bruneau J, Georgin-Lavialle S, Le Saché de Peufeilhoux L, Damaj G, Hadj-Rabia S (2015). Paediatric mastocytosis: a systematic review of 1747 cases. Br J Dermatol.

[128] Heinze A, Kuemmet TJ, Chiu YE, Galbraith SS (2017). Longitudinal study of pediatric Urticaria Pigmentosa. Pediatr Dermatol.

[129] Carter MC, Clayton ST, Komarow HD, Brittain EH, Scott LM, Cantave D (2015). Assessment of clinical findings, tryptase levels, and bone marrow histopathology in the management of pediatric mastocytosis. J Allergy Clin Immunol.

[130] Lozano AM, López JF, Zakzuk J, García E (2016). Papular urticaria: a review of causal agents in Colombia. Biomédica.

[131] Hernandez RG, Cohen BA (2006). Insect bite–induced hypersensitivity and the SCRATCH principles: a new approach to papular urticaria. Pediatrics.

[132] Jara LJ, Navarro C, Medina G, Vera-Lastra O, Saavedra MA (2009). Hypocomplementemic urticarial vasculitis syndrome. Curr Rheumatol Rep.

[133] Sag E, Tartaglione A, Batu ED, Ravelli A, Khalil SM, Marks SD, Ozen S (2014). Performance of the new SLICC classification criteria in childhood systemic lupus erythematosus: a multicentre study. Clin Exp Rheumatol.

[134] Krause K, Grattan CE, Bindslev-Jensen C, Gattorno M, Kallinich T, Koning HD (2012). How not to miss autoinflammatory diseases masquerading as urticaria. Allergy..

[135] Kuemmerle-Deschner JB, Ozen S, Tyrrell PN, Kone-Paut I, Goldbach-Mansky R, Lachmann H (2017). Diagnostic criteria for cryopyrin-associated periodic syndrome (CAPS). Ann Rheum Dis.

[136] Maurer M, Magerl M, Metz M, Siebenhaar F, Weller K, Krause K (2013). Practical algorithm for diagnosing patients with recurrent wheals or angioedema. Allergy..

[137] Youssef MJ, Chiu YE (2017). Eczema and urticaria as manifestations of undiagnosed and rare diseases. Pediatr Clin.

[138] Kozel MM, Mekkes JR, Bossuyt PM, Bos JD (1998). The effectiveness of a history-based diagnostic approach in chronic urticaria and angioedema. Arch Dermatol.

[139] Thomas P, Perkin MR, Rayner N, Cox H, Fox AT, Leech S (2008). The investigation of chronic urticaria in childhood: which investigations are being performed and which are recommended?. Clin Exp Allergy.

[140] Lang DM, Hsieh FH, Bernstein JA (2013). Contemporary approaches to the diagnosis and management of physical urticaria. Ann Allergy Asthma Immunol.

[141] Gimenez-Arnau A, Maurer M, De La Cuadra J, Maibach H (2010). Immediate contact skin reactions, an update of contact urticaria, contact urticaria syndrome and protein contact dermatitis–“a never ending story”. Eur J Dermatol.

[142] Krause K, Zuberbier T, Maurer M (2010). Modern approaches to the diagnosis and treatment of cold contact urticaria. Curr Allergy Asthma Rep.

[143] Hochstadter E. F., Ben-Shoshan M. (2013). Cold-induced urticaria: challenges in diagnosis and management. Case Reports.

[144] Caffarelli C, Dondi A, Povesi Dascola C, Ricci G (2013). Skin prick test to foods in childhood atopic eczema: pros and cons. Ital J Pediatr.

[145] Caffarelli C, Ricò S, Rinaldi L, Povesi Dascola C, Terzi C, Bernasconi S (2012). Blood pressure monitoring in children undergoing food challenge: association with anaphylaxis. Ann Allergy Asthma Immunol.

[146] Caffarelli C, Franceschini F, Caimmi D, Mori F, Diaferio L, Di Mauro D (2018). SIAIP position paper: provocation challenge to antibiotics and non-steroidal anti-inflammatory drugs in children. Ital J Pediatr.

[147] Caglayan Sozmen S, Povesi Dascola C, Gioia E, Mastrorilli C, Rizzuti L, Caffarelli C (2015). Diagnostic accuracy of patch test in children with food allergy. Pediatr Allergy Immunol.

[148] Sabroe RA, Fiebiger E, Francis DM, Maurer D, Seed PT, Grattan CE (2002). Classification of anti-FcϵRI and anti-IgE autoantibodies in chronic idiopathic urticaria and correlation with disease severity. J Allergy Clin Immunol.

[149] Sabroe RA, Grattan CE, Francis DM, Barr RM, Kobza Black A, Greaves MW (1999). The autologous serum skin test: a screening test for autoantibodies in chronic idiopathic urticaria. Br J Dermatol.

[150] Kilic G, Guler N, Suleyman A, Tamay Z (2010). Chronic urticaria and autoimmunity in children. Pediatr Allergy Immunol.

[151] Alyasin S, Karimi AA, Amiri A, Ehsaei MJ, Ghaffarpasand F (2011). Correlation between clinical findings and results of autologous serum skin test in patients with chronic idiopathic urticaria. Allergy Asthma Clin Immunol.

[152] Kulthanan K, Jiamton S, Gorvanich T, Pinkaew S (2006). Autologous serum skin test in chronic idiopathic Urticaria: prevalence, Cor-relation and clinical implications. Asian Pac. J Allergy Immunol.

[153] Fusari A, Colangelo C, Bonifazi F, Antonicelli L (2015). The autologous serum skin test in the follow-up of patients with chronic urticaria. Allergy.

[154] Grattan CEH, Wallington TB, Warin RP, Kennedy CTC, Lbradfield JW (1986). A serological mediator in chronic idiopathic urticaria—a clinical, immunological and histological evaluation. Br J Dermatol.

[155] Di Gioacchino M, Di Stefano F, Cavallucci E, Verna N, Ramondo S, Paolini F (2003). Treatment of chronic idiopathic urticaria and positive autologous serum skin test with cyclosporine: clinical and immunological evaluation. Allergy Asthma Proc..

[156] Schaefer P (2017). Acute and chronic Urticaria: evaluation and treatment. Am Fam Physician.

[157] Farkas H, Harmat G, Füst G, Varga L, Visy B (2002). Clinical management of hereditary angio-oedema in children. Pediatr Allergy Immunol.

[158] Potter P, Mitha E, Barkai L, Mezei G, Santamaría E, Izquierdo I, Maurer M (2016). Rupatadine is effective in the treatment of chronic spontaneous urticaria in children aged 2-11 years. Pediatr Allergy Immunol.

[159] Weller K, Groffik A, Magerl M, Tohme N, Martus P, Krause K (2013). Development, validation, and initial results of the angioedema activity score. Allergy..

[160] Ohanyan T, Schoepke N, Bolukbasi B, Metz M, Hawro T, Zuberbier T (2017). Responsiveness and minimal important difference of the urticaria control test. J Allergy Clin Immunol.

[161] Weller K, Groffik A, Church MK, Hawro T, Krause K, Metz M (2014). Development and validation of the Urticaria control test: a patient-reported outcome instrument for assessing urticaria control. J Allergy Clin Immunol.

[162] Sharma M, Bennett C, Cohen SN, Carter B (2014). H1-antihistamines for chronic spontaneous urticaria. Cochrane Database Syst Rev.

[163] Sánchez J, Zakzuk J, Cardona R (2016). Prediction of the efficacy of antihistamines in chronic spontaneous Urticaria based on initial suppression of the histamine-induced wheal. J Investig Allergol Clin Immunol.

[164] Fang S-Y, Perng D-W, Lee JY, Lin D-Y, Huangs CY (2010). An open-label, multicentre study of levocetirizine for the treatment of allergic rhinitis and urticaria in Taiwanese patients. Chin J Physiol.

[165] Fitzsimons R, van der Poel L-A, Thornhill W, Du Toit G, Shah N, Brough HA (2015). Antihistamine use in children. Arch Dis Child Educ Pract Ed.

[166] Hampel F, Ratner P, Haeusler J-M (2010). Safety and tolerability of levocetirizine dihydrochloride in infants and children with allergic rhinitis or chronic urticaria. Allergy Asthma Proc..

[167] Novák Z, Yáñez A, Kiss I, Kuna P, Tortajada-Girbés M, Valiente R (2016). Safety and tolerability of bilastine 10 mg administered for 12 weeks in children with allergic diseases. Pediatr Allergy Immunol.

[168] Johnson M, Kwatra G, Badyal DK, Thomas EA (2015). Levocetirizine and rupatadine in chronic idiopathic urticaria. Int J Dermatol.

[169] Simons FER (1999). Prospective, long-term safety evaluation of the H1-receptor antagonist cetirizine in very young children with atopic dermatitis. J Allergy Clin Immunol.

[170] Simons FER (2007). Safety of levocetirizine treatment in young atopic children: an 18-month study. Pediatr Allergy Immunol.

[171] Church MK, Maurer M, Simons FE, Bindslev-Jensen C, van Cauwenberge P, Bousquet J (2010). Global allergy and asthma European network. Risk of first-generation H(1)-antihistamines: a GA(2) LEN position paper. Allergy.

[172] Bousquet J, Khaltaev N, Cruz AA, Denburg J, Fokkens WJ, Togias A (2008). Allergic rhinitis and its impact on asthma (ARIA) 2008 update (in collaboration with the World Health Organization, GA(2) LEN and AllerGen). Allergy..

[173] Nolen TM (1997). Sedative effects of antihistamines: safety, performance, learning, and quality of life. Clin Ther.

[174] Hoxha M (2011). The treatment of severe urticaria with increasing doses of antihistamines. Allergy Eur J Allergy Clin Immunol.

[175] Grattan CEH, Humphreys F (2007). Guidelines for evaluation and management of urticaria in adults and children. Br J Dermatol.

[176] Staevska M, Popov TA, Kralimarkova T, Lazarova C, Kraeva S, Popova D (2010). The effectiveness of levocetirizine and desloratadine in up to 4 times conventional doses in difficult-to-treat urticaria. J Allergy Clin Immunol.

[177] Siebenhaar F, Degener F, Zuberbier T, Martus P, Maurer M (2009). High-dose desloratadine decreases wheal volume and improves cold provocation thresholds compared with standard-dose treatment in patients with acquired cold urticaria: a randomized, placebo-controlled, crossover study. J Allergy Clin Immunol.

[178] Abajian M, Curto-Barredo L, Krause K, Santamaria E, Izquierdo I, Church MK (2016). Rupatadine 20 mg and 40 mg are effective in reducing the symptoms of chronic cold Urticaria. Acta Derm Venereol.

[179] Finn AF, Kaplan AP, Fretwell R, Qu R, Long J (1999). A double-blind, placebo-controlled trial of fexofenadine HCl in the treatment of chronic idiopathic urticaria. J Allergy Clin Immunol.

[180] Nelson HS, Reynolds R, Mason J (2000). Fexofenadine HCl is safe and effective for treatment of chronic idiopathic urticaria. Ann Allergy Asthma Immunol.

[181] Church MK, Maurer M (2012). H1-antihistamines and urticaria: how can we predict the best drug for our patient?. Clin Exp Allergy.

[182] Weller K, Viehmann K, Bräutigam M, Krause K, Siebenhaar F, Zuberbier T, Maurer M (2013). Management of chronic spontaneous urticaria in real life--in accordance with the guidelines? A cross-sectional physician-based survey study. J Eur Acad Dermatol Venereol.

[183] Okubo Y, Shigoka Y, Yamazaki M, Tsuboi R (2013). Double dose of cetirizine hydrochloride is effective for patients with urticaria resistant: a prospective, randomized, non-blinded, comparative clinical study and assessment of quality of life. J Dermatol Treat.

[184] Staevska M, Gugutkova M, Lazarova C, Kralimarkova T, Dimitrov V, Zuberbier T (2014). Night-time sedating H1-antihistamine increases daytime somnolence but not treatment efficacy in chronic spontaneous urticaria: a randomized controlled trial. Br J Dermatol.

[185] Fedorowicz Z, van Zuuren EJ, Hu N (2012). Histamine H2-receptor antagonists for urticaria. Cochrane Database Syst Rev.

[186] Saini S, Rosen KE, Hsieh HJ, Wong DA, Conner E, Kaplan A (2011). A randomized, placebo-controlled, dose-ranging study of single-dose omalizumab in patients with H1-antihistamine-refractory chronic idiopathic urticaria. J Allergy Clin Immunol.

[187] Maurer M, Rosén K, Hsieh H-J, Saini S, Grattan C, Gimenéz-Arnau A (2013). Omalizumab for the treatment of chronic idiopathic or spontaneous urticaria. N Engl J Med.

[188] Saini SS, Bindslev-Jensen C, Maurer M, Grob JJ, Bülbül Baskan E, Bradley MS (2015). Efficacy and safety of omalizumab in patients with chronic idiopathic/spontaneous urticaria who remain symptomatic on H1 antihistamines: a randomized, placebo-controlled study. J Invest Dermatol.

[189] Kaplan A, Ledford D, Ashby M, Canvin J, Zazzali JL, Conner E (2013). Omalizumab in patients with symptomatic chronic idiopathic/spontaneous urticaria despite standard combination therapy. J Allergy Clin Immunol.

[190] Maurer M, Altrichter S, Bieber T, Biedermann T, Bräutigam M, Seyfried S (2011). Efficacy and safety of omalizumab in patients with chronic urticaria who exhibit IgE against thyroperoxidase. J Allergy Clin Immunol.

[191] Sussman G, Hébert J, Barron C, Bian J, Caron-Guay RM, Laflamme S, Stern S (2014). Real-life experiences with omalizumab for the treatment of chronic urticaria. Ann Allergy Asthma Immunol.

[192] Ensina LF, Valle SO, Juliani AP, Galeane M, Vieira dos Santos R, Arruda LK (2016). Omalizumab in chronic spontaneous Urticaria: a Brazilian real-life experience. Int Arch Allergy Immunol.

[193] Netchiporouk E, Nguyen CH, Thuraisingham T, Jafarian F, Maurer M, Ben-Shoshan M (2015). Management of pediatric chronic spontaneous and physical urticaria patients with omalizumab: case series. Pediatr Allergy Immunol.

[194] Ossorio-García L, Jiménez-Gallo D, Albarrán-Planelles C, Arjona-Aguilera C, Linares-Barrios M (2016). Chronic spontaneous urticaria in an 8-year-old girl treated with omalizumab. Clin Exp Dermatol.

[195] Boyce JA (2006). Successful treatment of cold-induced urticaria/anaphylaxis with anti-IgE. J Allergy Clin Immunol.

[196] Alba Marín JC, Martorell Aragones A, Satorre Viejo P, Gastaldo SE (2015). Treatment of severe cold-induced urticaria in a child with omalizumab. J Investig Allergol Clin Immunol.

[197] Kitsioulis NA, Xepapadaki P, Kostoudi S, Manousakis E, Douladiris N, Papadopoulos NG (2016). Omalizumab in pediatric cold contact urticaria: warm blanket for a cold bath?. Pediatr Allergy Immunol.

[198] Levi A, Tal Y, Dranitzki Z, Shalit M, Enk CD (2015). Successful omalizumab treatment of severe solar urticaria in a 6-year-old child. Pediatr Allergy Immunol.

[199] Neverman L, Weinberger M (2014). Treatment of chronic urticaria in children with antihistamines and cyclosporine. J Allergy Clin Immunol Pract.

[200] Doshi DR, Weinberger MM (2009). Experience with cyclosporine in children with chronic idiopathic urticaria. Pediatr Dermatol.

[201] Boubouka C, Charissi C, Koumintzis D, Kalogeromitros D, Stavropoulos P, Katsarou A (2011). Treatment of autoimmune urticaria with low-dose cyclosporin a: a one-year follow-up. Acta Derm Venereol.

[202] Savic S, Marsland A, McKay D, Ardern-Jones MR, Leslie T, Somenzi O, Baldock L, Grattan C (2015). Retrospective case note review of chronic spontaneous urticaria outcomes and adverse effects in patients treated with omalizumab or ciclosporin in UK secondary care. Allergy Asthma Clin Immunol..

[203] Asero R, Tedeschi A (2010). Usefulness of a short course of Oral prednisone in antihistamine-resistant chronic Urticaria: a retrospective analysis. J Investig Allergol Clin Immunol.

[204] Ledford D, Broder MS, Antonova E, Omachi TA, Chang E, Luskin A (2016). Corticosteroid-related toxicity in patients with chronic spontaneous urticaria. Allergy Asthma Proc.

[205] Di Lorenzo G, Pacor ML, Mansueto P, Esposito-Pellitteri M, Ditta V, Lo Bianco C, Leto-Barone MS, Di Fede G, Rini GB (2006). Is there a role for antileukotrienes in urticaria?. Clin Exp Dermatol.

[206] Nettis E, Colanardi MC, Paradiso MT, Ferrannini A (2004). Desloratadine in combination with montelukast in the treatment of chronic urticaria: a randomized, double-blind, placebo-controlled study. Clin Exp Allergy.

[207] De Silva NL, Damayanthi H, Rajapakse AC, Rodrigo C, Rajapakse S (2014). Leukotriene receptor antagonists for chronic urticaria: a systematic review. Allergy Asthma Clin Immunol.

[208] Sagi L, Solomon M, Baum S, Lyakhovitsky A, Trau H, Barzilai A (2011). Evidence for methotrexate as a useful treatment for steroid-dependent chronic urticaria. Acta Derm Venereol.

[209] Sharma VK, Singh S, Ramam M, Kumawat M, Kumar R (2014). A randomized placebo-controlled double-blind pilot study of methotrexate in the treatment of H1 antihistamine-resistant chronic spontaneous urticaria. Indian J Dermatol Venereol Leprol.

[210] Vena GA, Maurer M, Cassano N, Zuberbier T (2017). Alternative treatments for chronic spontaneous urticaria beyond the guideline algorithm. Curr Opin Allergy Clin Immunol.

[211] Shi CR, Li YP, Luo YJ, Shi CB, Yan X, Yang KH, Yi K (2012). IgE-mediated allergy: a rare cause of chronic spontaneous urticarial with allergen-specific immunotherapy as treatment option - a systematic review with meta-analysis from China. J Eur Acad Dermatol Venereol.

[212] Di Rienzo V, Cadario G, Grieco T, Galluccio AG, Caffarelli C, Liotta G (2014). Sublingual immunotherapy in mite-sensitized children with atopic dermatitis: a randomized, open, parallel-group study. Ann Allergy Asthma Immunol.

[213] Pajno GB, Bernardini R, Peroni D, Arasi S, Martelli A, Landi M (2017). Allergen-specific immunotherapy panel of the Italian Society of Pediatric Allergy and Immunology (SIAIP). Clinical practice recommendations for allergen-specific immunotherapy in children: the Italian consensus report. Ital J Pediatr.

[214] Pezeshkpoor F, Farid Hosseini R, Rafatpanah H, Shakerian B, Jabbari F, Zandkarimi MR (2012). Efficacy of atorvastatin and antihistamines in comparison with antihistamines plus placebo in the treatment of chronic idiopathic urticaria: a controlled clinical trial. Iran J Allergy Asthma Immunol.

[215] Kiyici S, Gul OO, Baskan EB, Hacioglu S, Budak F, Erturk E, Imamoglu S (2010). Effect of levothyroxine treatment on clinical symptoms and serum cytokine levels in euthyroid patients with chronic idiopathic urticaria and thyroid autoimmunity. Clin Exp Dermatol.

[216] Kocatürk E, Aktaş S, Türkoğlu Z, Kavala M, Zindanci I, Koc M, Can B, Südoğan S (2012). Autologous whole blood and autologous serum injections are equally effective as placebo injections in reducing disease activity in patients with chronic spontaneous urticaria: a placebo controlled, randomized, single-blind study. J Dermatolog Treat.

[217] Khafagy NH, Salem SAM, Ghaly EG (2013). Comparative study of systemic psoralen and ultraviolet a and narrowband ultraviolet B in treatment of chronic urticaria. Photodermatol Photoimmunol Photomed.

[218] Oguz Topal I, Kocaturk E, Gungor S, Durmuscan M, Sucu V, Yıldırmak S (2016). Does replacement of vitamin D reduce the symptom scores and improve quality of life in patients with chronic urticaria?. J Dermatol Treat.

[219] Long J, Wang Y, Pi X, Tu Y (2010). Clinical observation on the treatment of chronic urticaria with total glucosides of paeony capsule combined with citirizine. Chin J Integr Med.

[220] Wu CX, Li N, Xu ZH (2012). Effects of yiqi huoxue qufeng decoction on the diamine oxidase and immunoglobin E of patients with chronic urticaria. Zhongguo Zhong Xi Yi Jie He Za Zhi.

[221] Weller K, Koti I, Makris M, Maurer M (2013). Anxiety and depression seem less common in patients with autoreactive chronic spontaneous urticaria. Clin Exp Dermatol.

[222] Olsen JR, Gallacher J, Finlay AY, Piguet V, Francis NA (2016). Quality of life impact of childhood skin conditions measured using the Children’s dermatology life quality index (CDLQI): a meta-analysis. Br J Dermatol.

[223] Kuo CL, Chen CY, Huang HL, Chen WL, Lee HC, Chang CY (2014). Increased risk of major depression subsequent to a first-attack and non-infection caused urticaria in adolescence: a nationwide population-based study. BMC Pediatr.

[224] Infante M, Slattery MJ, Klein MH, Essex MJ (2007). Association of internalizing disorders and allergies in a child and adolescent psychiatry clinical sample. J Clin Psychiatry.

[225] Goh C, Lane AT, Bruckner AL (2007). Support groups for children and their families in pediatric dermatology. Pediatr Dermatol.

